# The GB4.0 Platform, an All-In-One Tool for CRISPR/Cas-Based Multiplex Genome Engineering in Plants

**DOI:** 10.3389/fpls.2021.689937

**Published:** 2021-07-01

**Authors:** Marta Vazquez-Vilar, Víctor Garcia-Carpintero, Sara Selma, Joan M. Bernabé-Orts, Javier Sanchez-Vicente, Blanca Salazar-Sarasua, Arianna Ressa, Carmine de Paola, María Ajenjo, Jose Carlos Quintela, Asun Fernández-del-Carmen, Antonio Granell, Diego Orzáez

**Affiliations:** ^1^Instituto de Biología Molecular y Celular de Plantas, Consejo Superior de Investigaciones Científicas-Universitat Politècnica de València, Valencia, Spain; ^2^Idoasis 2002 S.L., Madrid, Spain

**Keywords:** genome engineering, CRISPR/Cas, GoldenBraid, tobacco, multiplexing

## Abstract

CRISPR/Cas ability to target several loci simultaneously (multiplexing) is a game-changer in plant breeding. Multiplexing not only accelerates trait pyramiding but also can unveil traits hidden by functional redundancy. Furthermore, multiplexing enhances dCas-based programmable gene expression and enables cascade-like gene regulation. However, the design and assembly of multiplex constructs comprising tandemly arrayed guide RNAs (gRNAs) requires scarless cloning and is still troublesome due to the presence of repetitive sequences, thus hampering a more widespread use. Here we present a comprehensive extension of the software-assisted cloning platform GoldenBraid (GB), in which, on top of its multigene cloning software, we integrate new tools for the Type IIS-based easy and rapid assembly of up to six tandemly-arrayed gRNAs with both Cas9 and Cas12a, using the gRNA-tRNA-spaced and the crRNA unspaced approaches, respectively. As stress tests for the new tools, we assembled and used for Agrobacterium-mediated stable transformation a 17 Cas9-gRNAs construct targeting a subset of the Squamosa-Promoter Binding Protein-Like (SPL) gene family in *Nicotiana tabacum*. The 14 selected genes are targets of miR156, thus potentially playing an important role in juvenile-to-adult and vegetative-to-reproductive phase transitions. With the 17 gRNAs construct we generated a collection of Cas9-free *SPL* edited T_1_ plants harboring up to 9 biallelic mutations and showing leaf juvenility and more branching. The functionality of GB-assembled dCas9 and dCas12a-based CRISPR/Cas activators and repressors using single and multiplexing gRNAs was validated using a Luciferase reporter with the *Solanum lycopersicum* Mtb promoter or the *Agrobacterium tumefaciens* nopaline synthase promoter in transient expression in *Nicotiana benthamiana*. With the incorporation of the new web-based tools and the accompanying collection of DNA parts, the GB4.0 genome edition turns an all-in-one open platform for plant genome engineering.

## Introduction

Since the emergence of CRISPR/Cas as a genome editing tool in 2012, Cas site-specific nucleases have rapidly become central players in modern plant breeding due to their high precision with low propensity to off-target effects and the simplicity of their molecular machinery. CRISPR/Cas involves only a constant endonuclease element, typically the *Streptococcus pyogenes* Cas9 or the *Lachnospiraceae bacterium* Cas12a nuclease, and an easy-to-program variable element, a synthetic guide RNA (gRNA) in the case of Cas9, or a CRISPR RNA (crRNA) for Cas12a. Variable elements require only the change of a 20–23 nucleotides (nts) spacer sequence in order to determine its genomic target (Zhu et al., 2020). This simplicity explains the widespread use of CRISPR/Cas in many labs and small companies which have no access to previous editing tools e.g., zinc finger nucleases or transcription activator-like effector nucleases. Moreover, the ability of CRISPR/Cas to deliver multiple gRNAs simultaneously, a strategy known as multiplexing, enables targeting many loci at the same time. CRISPR/Cas multiplex editing in plant breeding paves the way for pyramiding favorable independent traits at unprecedented speed (Zhang et al., 2020). This capacity has been exemplified with the domestication of wild tomato by editing six genes involved in yield and productivity resulting in increased fruit size and number (Zsögön et al., 2018), or with its adaptation to urban agriculture by editing genes that resulted in compact tomato plants with precocious fruits (Kwon et al., 2020). In addition, multiplexing has the ability to uncover valuable traits which have remained elusive to breeding due to redundancy in large gene families. This is more evident in polyploid plants, which account for some of the most important crop species. Remarkable examples are low gluten wheat obtained upon mutation of 35 genes of the highly redundant α-gliadin family (Sánchez-León et al., 2018), glyco-engineered *Nicotiana benthamiana* plants with knockouts (KOs) in two xylosyl and four fucosyltransferase genes (Jansing et al., 2019) or semi-dwarf rapeseed with increased yield with biallelic mutations in the two *MORE AXILLARY GROWTH1* (*MAX1*) homeologue genes (Zheng et al., 2020).

In addition to genome editing, CRISPR/Cas is also becoming a popular tool for targeted gene activation and repression in plants (Lee et al., 2019; Papikian et al., 2019). This application relies on a nuclease-inactivated (dead) dCas-based programmable transcriptional regulator (PTR) and one or more gRNAs. Programmable regulators can be created by adding transcriptional regulatory domains, either Repressor Domains (RDs) or Activator Domains (ADs), that are jointly regarded as regulatory domains (RgDs) to a dCas. RgDs can be connected either directly as protein fusions or indirectly via a multiepitope peptide. In the latter case, multiple RgDs can be attached to the multiepitope peptide via a single chain antibody (scFv) intermediary [SunTag strategy (Tanenbaum et al., 2014)]. A more elaborated regulatory strategy consists in introducing modified gRNAs that incorporate RNA aptamers attached to different positions of the gRNA scaffold [SAM and scRNA strategies (Konermann et al., 2015)]. RNA aptamers specifically bind protein domains (e.g., the virus-derived MS2 domain) thus serving as anchoring points to MS2-fused ADs. We previously demonstrated that a modified scRNA strategy using a mutated RNA aptamer anchoring the VPR (VP64-p65-Rta) activation domain, resulted in a potent programmable activator tool in plants, able to selectively upregulate up to 10,000-fold the stress-inducible *Nicotiana benthamiana dihydroflavonol 4-reductase* (*DFR*) promoter (Selma et al., 2019).

Multiplex CRISPR/Cas constructs minimally involve three transcriptional units (TUs): (i) a plant selection marker, (ii) the Cas nuclease, and (iii) at least one gRNA. Additional gRNAs can be expressed either from several promoters as separate TUs, or from a single promoter as a polycistronic transcript that is further processed resulting in the active gRNAs. Cas9 has no ability to process gRNA tandem arrays, although recent studies in viral vectors seem to provide exceptions to this general rule (Uranga et al., 2021). Therefore, processable spacers need to be included in the array, so they can be rightly processed and trimmed into single functional units. Among the different spacer strategies described, the tRNA spacer method described by Xie et al. (2015) is being widely used in plant editing. This method relies in endogenous plant RNase P and RNase Z required to process the tRNAs flanking each spacer-scaffold unit. Other strategies that involve removable spacers include the Csy4 RNase (requiring the supply of the nuclease in trans), or a self-cleavable hammerhead ribozyme (Gao and Zhao, 2014; Cermák et al., 2017).

In the last decade, synthetic biology-inspired modular cloning strategies based on Type IIS restriction enzymes (REs), that cleave DNA outside of their recognition sites and allow the use of user-defined overhangs (Engler et al., 2009), have expanded our capacities for building combinatorial and multigene DNA constructs in binary vectors. The assembly of multiplex CRISPR/Cas constructs also benefits enormously from the capacity of Type IIS-based cloning methods such as Golden Gate (GG) to assemble large constructs containing several repeated elements, such as those conforming the gRNAs. Several GG-inspired cloning strategies enabling rapid construction of polycistronic, tRNA-spaced gRNA arrays have been developed over the past few years. Some of them rely on PCR amplification, thus requiring sequencing validation of the generated plasmids (Zuckermann et al., 2018), while others are PCR-free (Hahn et al., 2020; Oh et al., 2020). Although all these methods provide solutions for fast assembly of multiplex editing plasmids, none of them offer dedicated software tools for *in silico* design.

GoldenBraid (GB) is a well-established multigene engineering platform using standard Type IIS RE-based assembly rules (Sarrion-Perdigones et al., 2011). The most distinctive features of GB are the SynBio-inspired standardization of its DNA parts and the exchangeability of all GB-made constructs. GB assemblies involve three consecutive cloning steps. First, raw DNA sequences are adapted to the GB standard and cloned as Level 0 parts that are later assembled to create TUs or Level 1 parts. Owing to its iterative cloning strategy, any pair of Level 1 GB constructs can be straightforwardly assembled together with a Golden Gate reaction (Engler et al., 2009), greatly simplifying the creation of Level >1 complex multigene constructs. Successive versions of the system have extended its usability and its applications. Notably, GB version 3.0 incorporated a dedicated web that serves both as a software-assisted cloning tool as well as a repository of plant genetic elements comprising circa 800 public physical phytobricks and >14,000 user-exclusive virtual gene elements, including promoter regions, CDS, terminators, but also exchangeable transcriptional units and multigene constructs for e.g., conditional transgene expression, selection markers, etc. (Vazquez-Vilar et al., 2017). Each GB element is documented by a standard datasheet, which often incorporates functional (experimental) characterization. The GB platform has provided solutions to challenging multigene engineering endeavors as the engineering of the twelve-gene cholesterol pathway in Arabidopsis (Sonawane et al., 2016), the three genes betalain pathway in tomato (Polturak et al., 2016) or the engineering of a memory switch in *N. benthamiana* (Bernabé-Orts et al., 2020).

In the advent of the plant genome engineering era, we launched this fourth version of GB, GB4.0 genome edition, that aims to offer extended capabilities to facilitate genome engineering, taking advantage of the easy cloning and its software integration. First, we aimed to simplify the assembly of constructs for multiplex genome engineering in plants by providing, in addition to a full set of functionally validated vectors, a software-assisted cloning procedure. To do so, we incorporated to the GB platform new software tools devoted to CRISPR/Cas construct-making, thus generating a new multipurpose bioengineering resource for plants that exploits the synergies between CRISPR/Cas tools on one side, and modular cloning and Synthetic Biology on the other side. Over the last 5 years we have enriched the GB DNA parts repository incorporating (i) all elements required for Cas9 and Cas12a single-guide and multiplex editing, plus (ii) new DNA parts and constructs required for dead Cas9 (dCas9)-based programmable gene regulation, also including DNA elements for multiplex targeting. The GB4.0 genome edition platform described here contains new software tools so that all guide RNA cloning procedures, including polycistronic Cas9 and Cas12a gRNAs, are fully software-assisted, generating output files with detailed laboratory protocols and annotated Genbank constructs. Taking advantage of the features of the cloning system described above, the operative limits of some of the most useful GB4.0 tools were investigated. In a first example, we tested a large 17 tRNA-spaced gRNAs multiplex construct designed to knock-out 14 genes belonging to the *Nicotiana tabacum Squamosa-Promoter Binding Protein-Like* (*SPL*) gene family. This experiment generated a highly diverse population of T_0_ mutant plants, some of which accumulated up to five biallelic and four heterozygous mutations in as many homolog genes and leading to transgene-free T_1_ plants with up to nine biallelic mutations. In a separate example, we tested the ability of dCas9-based programmable transcriptional activator to super-activate a strong plant promoter in *N. benthamiana*, yielding expression levels well above those of the constitutive CaMV 35S promoter. Noteworthy, and owing to its initial design as a community collaborative tool, GB4.0 offers all users the possibility to easily exchange their newly developed constructs/tools with the community, promoting a cooperation and democratization in new breeding techniques.

## Materials and Methods

### Guide RNA Assembly on Level 0 and Level 1 Plasmids

All Cas9 gRNAs used in this work with editing purposes were assembled on Level 0 plasmids following the procedure described on Supplementary Figure 1 and using the tools listed in the Supplementary Table 1 for Cas9 multiplexing. Briefly, for the assembly of gRNAs on level 0, two partially complementary primers designed at https://gbcloning.upv.es/do/crispr/multi_cas9_gRNA_domesticator_1 using as input the sequences listed in Supplementary Table 2, were included in a BsmBI restriction–ligation reaction together with the pUPD2 and the corresponding level−1 tRNA-scaffold plasmid depending on the desired position of each target on the level 1 assembly. Level−1 tRNA-scaffold plasmid selection and assembly planning was done at https://gbcloning.upv.es/do/crispr/multi_cas9_gRNA_domesticator_2/. Cas9 gRNAs used for activation were assembled as Level 1 constructs following the single gRNA strategy (Supplementary Figure 1) using partially complementary primers designed at https://gbcloning.upv.es/do/crispr/Single_Cas9_gRNA_Domesticator. All gRNA constructs were validated by RE-analysis and Sanger sequencing.

Cas12a crRNAs were assembled following the procedures described in Supplementary Figure 1 and using the tools listed in the Supplementary Table 1 for Cas12a single and multiplexing crRNAs. Briefly, for Cas12a single crRNAs two partially complementary primers were designed at https://gbcloning.upv.es/do/crispr/Single_Cas12a_gRNA_Domesticator using as input the sequences listed in Supplementary Table 2 and included in a BsaI restriction-ligation reaction together with GB1443, GB1444, and a Level 1 destination vector. For the assembly of the Cas12a 3X crRNA, a synthetic DNA fragment was designed at https://gbcloning.upv.es/tools/cas12multiplexing_domestication/, purchased from GenScript and cloned as a Level 0 DNA part prior to its assembly with GB1443 in a Level 1 plasmid. All crRNA plasmids were validated by RE-analysis and Sanger sequencing.

All plasmids required for gRNA/crRNA assemblies with GB are listed in Supplementary Table 3 and available at Addgene (https://www.addgene.org/).

### Cloning in α and Ω-Level Destination Vectors

GB multigenic assemblies rely on the use of a set of four destination vectors (α1, α2, Ω1, Ω2) as previously described in Sarrion-Perdigones et al. (2011). Briefly, α-level plasmids are designed for Level 1 multipartite assemblies of Level 0 parts with BsaI. The Ω-level vectors allow binary assemblies with BsmBI of Level 1 TUs or Level >1 modules assembled in compatible (1 and 2) α-level vectors. In the same way, and closing a loop, Level >1 modules assembled in compatible (1 and 2) Ω-level vectors can be assembled in any α-level vector with BsaI. All α-level and Ω-level vectors used in this study have a pCAMBIA backbone.

Multipartite BsaI restriction–ligation reactions from Level 0 parts and binary BsaI or BsmBI restriction–ligation reactions were performed as described in Vazquez-Vilar et al. (2020) to obtain all the Level ≥1 assemblies. All Level ≥1 plasmids were validated by RE-analysis.

The sequences of all Level ≥1 constructs used in this study can be found entering their IDs (displayed at Supplementary Table 4) at https://gbcloning.upv.es/search/features/.

### Plant Material

The *N. benthamiana* laboratory strain was used for all transient expression assays. *N. tabacum* cv. K326 was used for stable transformation. A line previously developed in the laboratory using CRISPR/Cas9 with biallelic homozygous mutations in six *SPL* genes and biallelic heterozygous mutations in one *SPL* gene (SPL157-5 T_1_) was used as genetic background for retransformation after T-DNA segregation. Edited genes and mutations of the SPL157-5 T_1_ line are: Nitab4.5_0003348g0050.1 (213delC), Nitab4.5_0007487g0020.1 (204delCA), Nitab4.5_0002219g0060.1 (213insA), Nitab4.5_0000638g0040.1 (559delGGACACAA/565delAA), Nitab4.5_0001752g0040.1 (353delACAAC), Nitab4.5_0003572g0010.1 (289insC), Nitab4.5_0000016g0300.1 (535insT). In between brackets number indicates the position of the mutation in reference to the ATG, “del” states for deletion, “ins” states for insertion. It should be noticed that GB2714, include one gRNA (gSPL3.1) targeting Nitab4.5_0003348g0050.1 at a position different to that mutated on plant SPL157-5. Therefore, editing efficiencies of this gRNA targeting this specific gene were also considered for calculating the data presented in section GB-Made Multiplex Constructs Facilitate Editing of Gene Families in Tobacco.

### Plant Transformation

Transient expression assays in *N. benthamiana* plants were carried out as previously described by Vazquez-Vilar et al. (2017). Briefly, overnight *Agrobacterium tumefaciens* (renamed to *Rhizobium radiobacter*) strain GV3101 cultures were pelleted and resuspended in agroinfiltration buffer (10 mM MES, pH 5.6, 10 mM MgCl_2_, and 200 μM acetosyringone). Bacterial suspensions were incubated for 2 h at room temperature on a horizontal rolling mixer and their optical density at 600 nm (OD_600_) was adjusted to 0.1. Equal volumes of bacterial suspensions were mixed for experiments in which more than one GB element was used. Agroinfiltrations were carried out through the abaxial surface of the three youngest leaves of each plant with a 1 ml needle-free syringe. The silencing suppressor P19 was included in all the assays, either in the same T-DNA for the transcriptional regulation experiments or co-delivered in an independent T-DNA for the targeted mutagenesis assays.

For *N. tabacum* cv. K326 stable transformation, *A. tumefaciens* LBA4404 harboring plasmid GB2714 was used. Transformation was performed following a standard protocol (Horsch et al., 1985). Briefly, fully expanded leaves of SPL157-5 T_1_ were sterilized with 5% commercial bleach (40 g of active chlorine per liter) for 10 min followed by four consecutive washing steps with sterile demi-water. Leaf discs (*d* = 0.8 cm) were cut with a cork borer and incubated overnight in co-culture plates [4.9 g/L Murashige and Skoog medium (MS) supplemented with vitamins (Duchefa), 3% sucrose (Sigma-Aldrich), 0.9% Phytoagar (Duchefa), 1 mg/L BAP (Sigma-Aldrich), 0.1 mg/L NAA (Sigma-Aldrich), pH 5.7]. Leaf discs were incubated for 15 min with the Agrobacterium culture (OD_600_ = 0.3). Then, the discs were returned to the co-culture plates and incubated for 2 days in darkness. Next, discs were transferred to selection medium [4.9 g/L MS supplemented with vitamins (Duchefa), 3% sucrose (Sigma-Aldrich), 0.9% Phytoagar (Duchefa), 1 mg/L BAP (Sigma-Aldrich), 0.1 mg/L NAA (Sigma-Aldrich), 500 mg/L carbenicillin, 100 mg/L kanamycin, pH 5.7]. Discs were transferred to fresh medium every seven days until shoots appeared (4–6 weeks). Shoots were cut and transferred to rooting medium [4.9 g/L MS supplemented with vitamins (Duchefa), 3% sucrose (Sigma-Aldrich), 0.9% Phytoagar (Duchefa), 500 mg/L carbenicillin, 100 mg/L kanamycin, pH 5.7] until roots appeared. Growing conditions were in all steps 16 h light/8 h dark, 25°C, 60–70% humidity, 250 μmol m^−2^ s^−1^ photons.

### Genomic DNA Extraction and Editing Efficiency Evaluation

Each 150 mg of either leaf material from T_0_ tobacco stable transformants or from 5 days post infiltration (dpi) *N. benthamiana* leaves was used for genomic DNA extraction. Genomic DNA was extracted with the cetyl trimethylammonium bromide (CTAB) method (Murray and Thompson, 1980). The genomic regions flanking the nuclease target sites were PCR amplified using MyTaq^TM^ DNA Polymerase (Bioline) and primers listed in Supplementary Table 5. The PCR amplicons were confirmed on a 1% agarose gel electrophoresis and purified with ExoSAP-IT™ PCR Product Cleanup Reagent (ThermoFisher Scientific) following the manufacturer's indications prior to Sanger sequencing. Chromatograms of Cas9-edited genomic DNA were analyzed using Inference of CRISPR Edits (ICE) v2 tool from Synthego (https://ice.synthego.com/) and chromatograms of Cas12a-edited genomic DNA were analyzed with TIDE (http://shinyapps.datacurators.nl/tide/). All analyses were manually curated. When two or more gRNAs targeting the same gene show a 50% of editing efficiency for each gRNA it is not possible to elucidate if one or both alleles are mutated. On these cases, estimations were done in the most conservative way and heterozygous mutations were assumed.

### Sampling and Luciferase Assays

Samples of leaves coinfiltrated with the *A. tumefaciens* nopaline synthase promoter (pNos) or the *Solanum lycopersicum* Mtb promoter (pMtb) reporter construct (GB1116 or GB1399, respectively), different activator/repressor TUs or modules (GB1830, GB1190, GB1826, GB2047, GB1668) and the individual or polycistronic gRNAs targeting either the pNos or the pMtb (Supplementary Tables 2, 4) were collected at 4 dpi. Firefly luciferase (Fluc) and *Renilla reniformis* luciferase (Rluc) activities were determined as previously described by Vazquez-Vilar et al. (2017). Fluc/Rluc ratios were determined as the mean value of three samples coming from three independent agroinfiltrated leaves of the same plant and were normalized to the Fluc/Rluc ratio obtained for a reference sample including the pNos reporter GB1116. GB1119, a CaMV 35S reporter construct was included as upper limit reference in all experiments.

## Results

### Setup of the General GB4.0 Cloning Pipeline for Genome Editing

**Figure 1:**
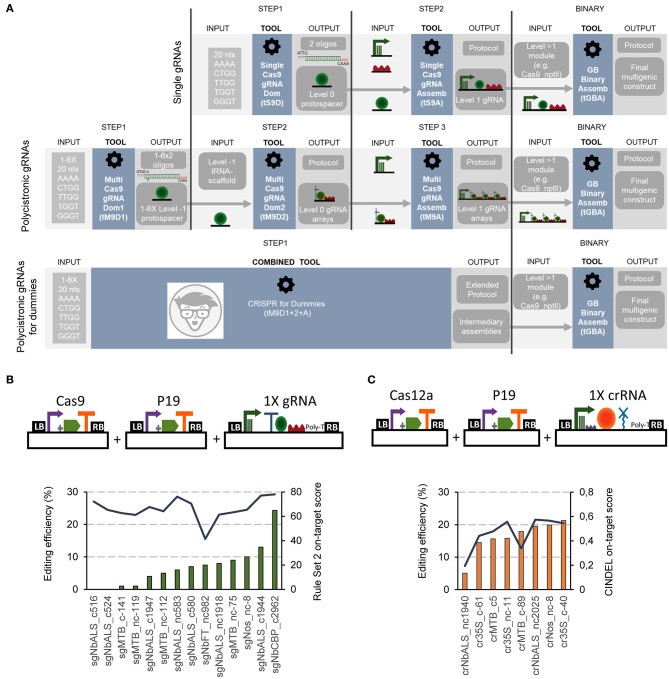
Flowchart and functional validation of the software-assisted procedure for gRNAs cloning with GoldenBraid. **(A)** Schematic representation of the software tools for Cas9 gRNAs cloning. Single Cas9 gRNAs cloning is assisted via the *Single Cas9_gRNA Domesticator* (*tS9D*) and the *Single Cas9_gRNA Assembler* (*tS9A*) (top). Cas9 multiplexing gRNAs are assembled with the use of three consecutive software tools: the *Multi Cas9_gRNA Domesticator 1* (*tM9D1*), the *Multi Cas9 gRNA Domesticator 2* (*tM9D2*), and the *Multiple Cas9_gRNA Assembler* (*tM9A*) that creates Level 1 gRNA arrays (middle). The *CRISPR for Dummies* tool takes 1–6 protospacers as input and generates Level 1 gRNA arrays in a single step (bottom). **(B)** Correlation of Cas9 gRNAs predicted on-target scores and editing efficiencies tested in *N. benthamiana* transient expression. Schematic representation of the plasmids co-infiltrated in this experiment (top) and Cas9 guide RNAs editing efficiencies (left axis, bars, determined with ICE) and their corresponding on-target score (right axis, line) determined with the “Rule Set2 scoring” (bottom). **(C)** Correlation of Cas12a crRNAs predicted on-target scores and editing efficiencies tested in *N. benthamiana* transient expression. Schematic representation of the plasmids co-infiltrated in this experiment (top) and Cas12a guide RNAs editing efficiencies (left axis, bars, determined with TIDE) and their corresponding on-target score (right axis, line) determined with CINDEL (bottom).

GB cloning operates at three hierarchically organized assembly levels (Supplementary Figure 1): Level 0 parts are basic elements such as promoters, coding sequences (CDSs), terminators, etc., which can be assembled using multipartite Type IIS restriction-ligation reactions with BsaI to create level 1 elements, usually TUs. Level 1 elements are then combined binarily also via Type IIS restriction-ligation reactions to create higher order multigene structures (named level >1 elements), following an iterative cloning pipeline that alternates the use of BsaI and BsmBI as Type IIS REs. A basic CRISPR/Cas genome editing construct comprises at least three level 1 TUs: a TU containing the guide RNA(s), a Cas-expressing TU, and a third unit usually encoding a selection marker. From a modular cloning perspective, the most distinct element is the gRNA unit. For the expression of gRNAs, the RNA Pol III promoters, which specifically transcribe small nuclear RNAs in the cell, are preferably used. The gRNA TU can be designed to express just one gRNA (single RNA strategy), or it can be engineered to contain several tandemly arrayed gRNAs under the control of a single (usually Pol III) promoter (polycistronic strategy). In both cases, subsequent GB cloning iterations allow the assembly of additional gRNA TUs (single or polycistronic) to expand the multiplexing capacity. Specific software tools were developed for the design and assembly of single or polycistronic gRNAs, both for Cas9 and Cas12a (see Figure 1A; Supplementary Table 1 and https://gbcloning.upv.es/tools/grna/). We created independent strategies for cloning single and polycistronic guide RNA constructs both for Cas9 and Cas12a due to the distinct features of these two endonucleases: (i) the native constant elements of the Cas12a guide RNA are located at the 5'end, whereas the spacer is located at the 3'end opposite to Cas9; (ii) most efficient Cas12a spacers are 23 nts length, while optimum Cas9 spacers have 20 nts, and (iii) Cas12a is able to self-process a polycistronic RNA, while Cas9 needs additional signals. Prior to gRNA assembly, users need to find convenient protospacers for targeting the desired gene(s), using for this purpose external tools as those suggested as links in the GB webpage [i.e., Benchling (https://www.benchling.com/), CRISPOR (Concordet and Haeussler, 2018), CINDEL (Kim et al., 2017), CRISPR-DT (Zhu and Liang, 2019)].

### Software-Assisted Cloning and Functional Test of Single Guide RNA Constructs

For the assembly of single gRNA constructs for Cas9 editing, a two-steps procedure was established (Figure 1A upper panel; Supplementary Figure 1). In a first step, the user-selected spacer sequence (20 nts) is introduced as input into the *Single Cas9_gRNA domesticator tool* (*tS9D*). The *tS9D* produces an output consisting in a pair of overlapping oligonucleotides containing the spacer sequence itself, plus the appropriate cloning overhangs (step 1). It is important to mention that the two custom oligos designed by the *tS9D* are the only elements in the whole cloning procedure that are not supplied by the GB collection. A second step, assisted by the *Single Cas9_gRNA Assembler tool* (*tS9A*) takes the pair of oligonucleotides designed by the *tS9D* as input and combines them in a BsaI restriction-ligation reaction (step 2) with a Pol III promoter and the Cas9 scaffold, both elements provided in the collection. This second webtool also provides a detailed protocol of the assembly reaction.

The single Cas12a crRNA GB cloning comprises also two steps (Supplementary Figure 1). In the first one, a new level 0 element is designed using the *Single Cas12a_crRNA domesticator (tS12D)*. This tool takes a 20–23 nts spacer input and designs a pair of overlapping oligonucleotides containing this sequence and the appropriate overhangs for cloning. In a second step, the *Single Cas12a_crRNA assembler* (*tS12A*) combines the *tS12D* output with the remaining level 0 elements, namely an upstream constant element comprising the Pol III promoter and the crRNA direct repeats (DRs), and the HDV ribozyme that will trim the 3' end to expose the last nt of the spacer.

We functionally tested the new tools by assembling both Cas9 and Cas12a editing constructs targeting transgenic (i.e., the CaMV 35S, *A. tumefaciens* pNos and *Solanum lycopersicum* pMtb promoters) and endogenous sequences (i.e., XT, FT, CBP, and ALS) in *N. benthamiana*. Besides construct checking, we used this test to evaluate two earlier described gRNA efficiency algorithms, one for Cas9 gRNAs (the Rule Set 2 scoring algorithm, Doench et al., 2016), and another for Cas12a (CINDEL, Kim et al., 2017). We co-infiltrated the assembled gRNAs/crRNAs along with the Cas9 or the Cas12a driven by the CaMV 35S promoter. To assess mutation frequencies, the target site was PCR-amplified from genomic DNA, Sanger sequenced and analyzed using Synthego. The observed editing efficiencies ranged from 0 to 24% for Cas9 gRNAs (Figure 1B) and from 5 to 21% for Cas12a crRNAs (Figure 1C). We found a correlation coefficient of 0.82 between experimentally determined editing efficiencies and CINDEL predicted scores for Cas12a crRNAs, while for Cas9 gRNAs we could not detect any correlation between the predicted scores and experimental efficiencies.

### Software-Assisted Cloning of Polycistronic Guide RNA Constructs

To create polycistronic gRNAs for Cas9 editing, the GB pipeline uses tRNAs as spacers, which are later processed in planta by endogenous RNases (Xie et al., 2015). Three software tools, depicted in Figure 1A (central panel), guide researchers through a three-steps cloning process: (i) individual spacers are domesticated and added to the database (assisted by the *Multiple Cas9_gRNA domesticator tool 1, tM9D1*); (ii) level 0 parts (individual gRNAs) are constructed, each comprising a tRNA, the previously domesticated spacers and a scaffold element, all three assembled in a BsmBI reaction (*Multiple Cas9_gRNA domesticator tool 2, tM9D2*); (iii) a polycistronic gRNA is assembled combining up to six level 0 gRNAs from step 2, plus a Pol III promoter (*Multiple Cas9_gRNA assembler, tM9A*). Again, all elements except the spacer oligos are available in the collection. Researchers only need to decide beforehand (step 2) the number of gRNAs (from one to six) that will conform the array and select the level 0 parts to be created accordingly.

If a multiple Cas12a crRNA design is selected, the process will comprise two steps, assisted by the *Multiple Cas12a_crRNA domesticator (tM12D) and the Multiple Cas12a_crRNA assembler (tM12A)*, respectively (see Supplementary Figure 1). First, the *tM12D* takes as input two to six 20–23 nts spacer sequences and designs a tandem of scaffold-spacer units flanked by BsmBI sites as an output. It should be noted that the output of the *tM12D* tool is a >200–500 bp DNA fragment, which needs to be produced via chemical synthesis and assembled as a level 0 part. Next, the *tM12A* combines the *tM12D* output with an additional level 0 element including the Pol III promoter and the first crRNA DRs, which is the same as that used by the *tS12A* tool. In this sense, Cas12a polycistronic crRNA requirements are more demanding in terms of DNA synthesis than Cas9, where only a pair of overlapping oligos was required.

Once a single or multiplexed level 1 Cas9 or Cas12a guide RNA TU is created, this can be combined with other TUs using the regular *GB Binary assembly* (*BA*) tool. For convenience, the public GB collection contains a number of frequently used pre-made TUs and multigene modules, such as Cas9 TUs, Cas12a TUs, negative and positive selection markers, and combinations of those (e.g., GB0639, GB2234, GB1441, GB2085, GB3819, etc.). Several gRNA TUs (single or polycistronic) can be combined in the same binary fashion to create large “two dimensions” multiplexing arrays. Detailed lists of recommended GB TUs and modules for each application, namely gene editing, gene activation or gene repression either with Cas9 or with Cas12a can be accessed at https://gbcloning.upv.es/tools/crispr/.

### Fast-Track Cas9 Multiplex Assembly Tool

To further facilitate the assembly of Cas9 multiplexing constructs, a simplified tool combining the three above described Cas9 multiplexing tools was created, nicknamed “*CRISPR for Dummies*” (Figure 1A, lower panel; https://gbcloning.upv.es/do/crispr/cas9_multiplexing/crispr_for_dummies/). In this tool, input choices are reduced to the number (N) of arrayed gRNAs to be assembled (from one to six), and the sequences of the 20 nt spacers to be used. As an output, user's obtain three sets of information: (i) the sequence of the 2xN oligos for synthesis; (ii) a detailed laboratory protocol where all GB elements to be used in the assembly are included, (iii) Genbank and SBOL files of all intermediate plasmids, and (iv) a Genbank file of the final construct comprising: a multiplexed gRNA TU, a constitutively expressed Cas9, an nptII gene for positive selection, and a DsRed gene for both positive selection in the T_0_ and negative selection in the T_1_.

All described software-tools, listed in Supplementary Table 1, were conceived to provide a centralized user-friendly gateway for the design of CRISPR/Cas constructs, both for genome editing and for other expanded applications as gene regulation. All GB-software tools provide Genbank output files next to a vSBOL-based representation of the generated construct (Baig et al., 2021) and a detailed protocol. All CRISPR/Cas constructs are assembled in destination binary plasmids ready for Agrobacterium-mediated transformation. Typically, T_0_ transgenic plants are genotyped by PCR to assess the edition levels in the target loci, and plants showing highest edition levels are self-pollinated to the next generation, where T-DNA free T_1_ plants are re-evaluated for the intended phenotype. The inclusion of a fluorescent protein TU in the final construct, e.g., DsRed, facilitates the selection of T-DNA-free T_1_ plants.

### GB-Made Multiplex Constructs Facilitate Editing of Gene Families in Tobacco

**Figure 2:**
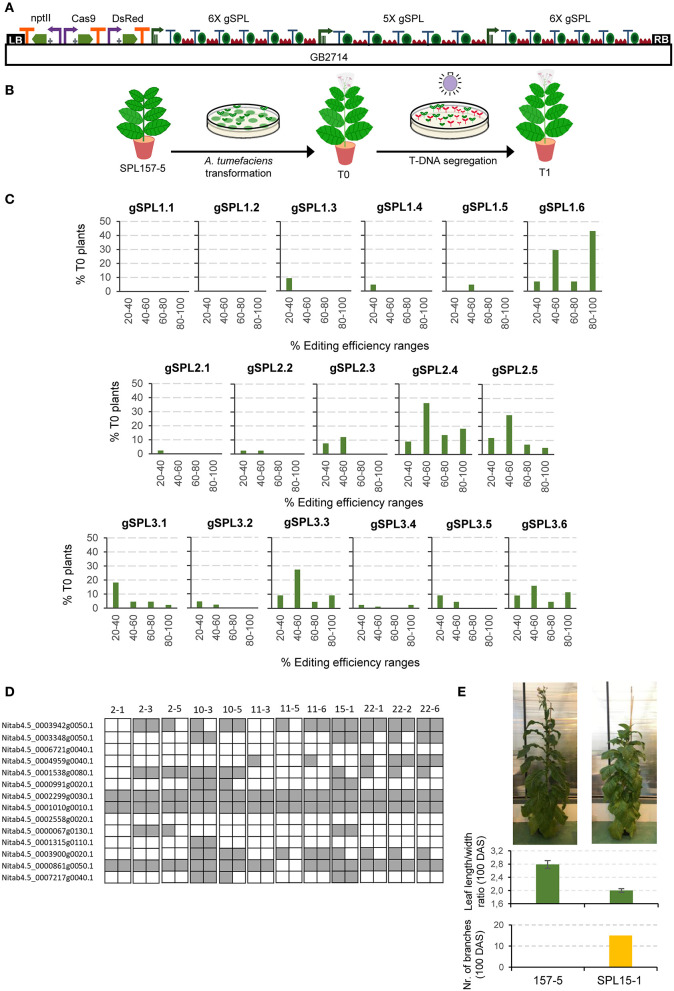
Cas9 guide RNAs editing efficiencies in tobacco stable transformation. **(A)** Construct including 17 gRNAs (6X+5X+6X) targeting *SPL* genes that was used for *A. tumefaciens* transformation. **(B)** Schematic representation of tobacco transformation with *A. tumefaciens*. **(C)** gRNA efficiency for each position in the polycistron evaluated for 22 T_0_ tobacco plants by jointly analyzing the data obtained for all genes targeted by the same gRNA. **(D)** Schematic representation of a selection of TDNA-free T_1_ *SPL* edited lines. Each square represents an allele. Gray indicates edited alleles while white represents wild type alleles. **(E)** Pictures of a T_1_ *SPL* edited plant (SPL15-1) and a control plant (157-5) taken 100 days after sowing (DAS). Average leaf length/width ratio and number of branches at 100 DAS. Error bars represent standard deviation of individual leaves of the same plant (*n* = 5).

As a stress-test for functionality of GB4.0 tools, we cloned and assayed a 17 gRNAs Cas9 multiplex construct aimed at mutagenizing a subset of 14 members of the large *SPL* transcription factors family in tobacco. The final editing construct, named GB2714 (depicted in Figure 2A), comprises a nptII positive selection marker, a Cas9 TU, a DsRed TU used as an additional positive selection marker in the T_0_ and as a negative marker in the T_1_, and three arrays of 6X, 5X, and 6X gRNAs respectively directed to different positions of the *SPL* genes targets (Supplementary Figure 2). The GB2714 construct was transformed in a plant line (SPL157-5 T_1_) of *N. tabacum* cv. K326 which had been previously mutagenized in a different subset of genes in the *SPL* family (Willmann, 2021, in press) (Figure 2B). The resulting 22 T_0_ regenerated plants were analyzed by ICE (Synthego) to estimate the editing efficiency of each individual gRNA, as summarized in Figure 2C.

To better visualize possible influences of positional effects in the array, editing efficiencies estimates determined for the 22 T_0_ plants were grouped in four ranges, with only rates above 20% considered as positive. Mutation estimations between 40 and 60% were presumed as heterozygous mutations; rates estimated above 80% were regarded as biallelic, and the remaining intermediate levels were considered as chimeric affecting one (20–40%) or both (60–80%) alleles. As expected, edition estimates show highly variable values, with two gRNAs (gSPL1.1 and gSPL1.2) showing values below the threshold, whereas others as gSPL1.6 showed 43% putative biallelic rates. It is important to note that all positions in both 5X and 6X arrays yielded estimates above the 20% threshold for at least one of the gRNAs assayed, indicating that all array positions are active. However, our data suggest some positional bias in the editing efficiency. In the three polycistronic arrays assayed, biallelic mutations were recovered for targets whose gRNA was in the last position (from 5% in gSPL2.5 to 43% in gSPL1.6). In contrast, only one gRNA set in position 1 (gSPL3.1) was able to produce biallelic mutatnts, and only in a small proportion (2%). In general, the last position turned out to be the most effective in two of the three polycistronic arrays, whereas in the third array position 4 was first in the ranking of efficiency.

The described strategy successfully pyramided multiple knock-outs in T_0_ and T_1_ generations, the latter easily made transgene free by DsRed-negative selection of T_1_ seeds grown *in vitro* (later tested by PCR, data not shown). The highest mutation rates obtained in T_0_ corresponded to a plant having six biallelic and three heterozygous mutations. Figure 2D and Supplementary Table 6 display the genotype of 12 T-DNA-free T_1_ plants derived from the five T_0_ selected lines. All plants show a distinctive phenotype, including delayed flowering time, more branching and extended juvenility. As an example, plant SPL15-1 is shown. This plant had 15 branches and an average leaf length/width ratio of 2.0 at 100 days after sowing, while the control line evaluated at the same time did not present any branches and had a leaf length/width ratio of 2.8 (Figure 2E). A decreased length/width ratio in tobacco leaves is known to be related with juvenility (Feng et al., 2016). The best T-DNA-free T_1_ line showed up to nine biallelic and one heterozygous mutations in as many *SPL* genes (plant SPL10-3). As mentioned above, the present experiment was performed on top of a previously mutagenized T_1_ plant harboring six biallelic mutations in a different subset of SPL genes, therefore the resulting genotype of SPL10-3 comprised 15 KOs out of the circa 28 miRNA156-regulated *SPL* genes in *N. tabacum*.

### Beyond DNA Cuts: GB-Made dCas9 and dCas12a-Based Programmable Regulators for Gene Activation and Repression

GB4.0 also incorporates webtools and DNA elements for the design of Cas9 and Cas12a-based PTRs.

In both direct dCas-RgD and indirect dCas-SunTag plus scFv-RgD examples, the design of a gRNA TU remains the same as in the previously described editing constructs. The only difference in the design pipeline takes place during binary assemblies, where transcriptional units conveniently equipped with dCas-RgD or dCas-SunTag plus scFv-RgD will be incorporated instead of the regular Cas9 endonuclease. Several dCas-RgD and dCas9-SunTag fusions are available in the GB public repository, together with different scFv-RgD fusions to complement the SunTag strategy (https://gbcloning.upv.es/tools/crispr/regulation/TUs/).

**Figure 3:**
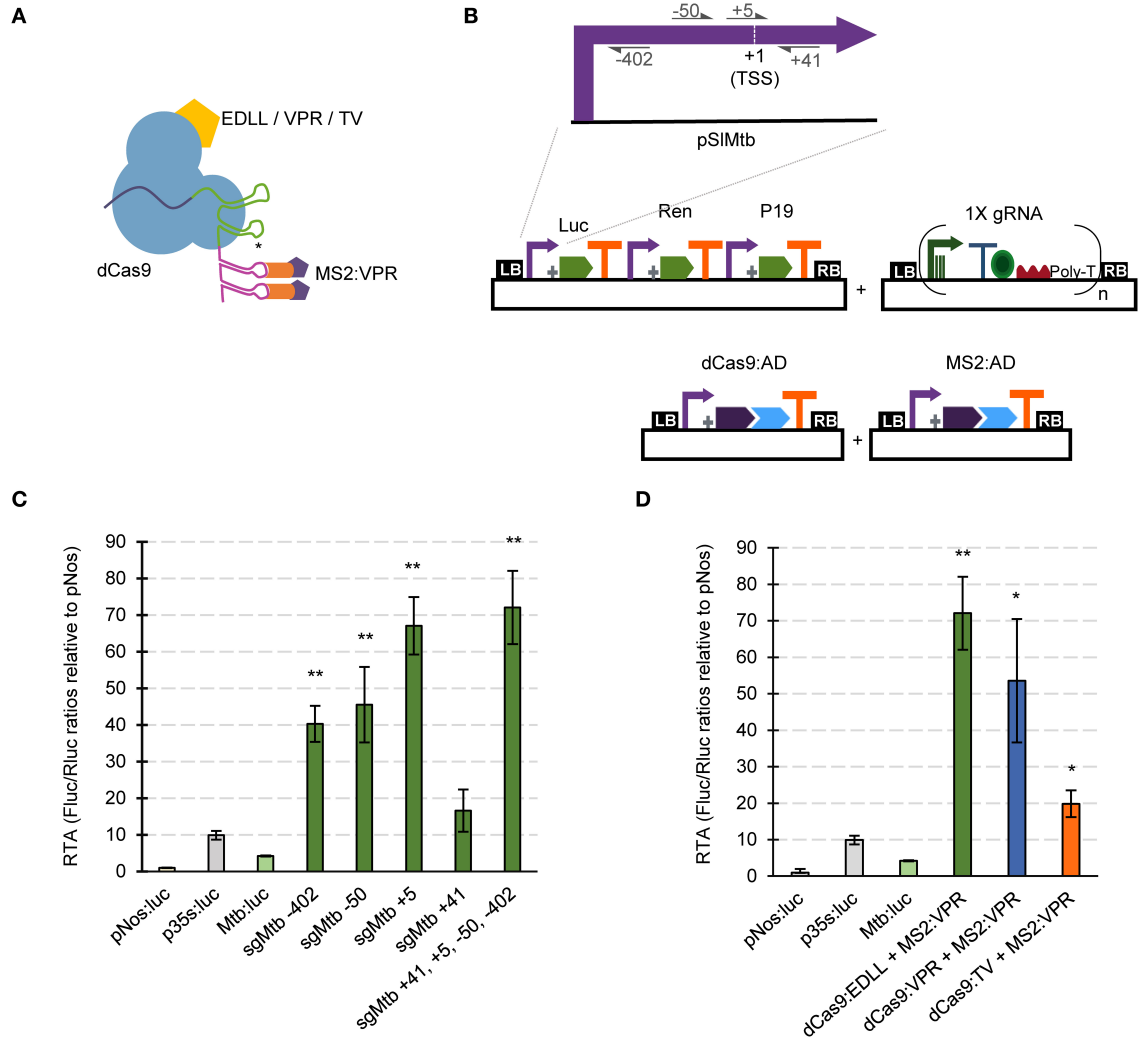
dCas9-based positive regulation in transient expression in *N. benthamiana*. **(A)** Schematic representation of the GB plasmids co-infiltrated for evaluating dCas9-scRNA for activation of the *Solanum lycopersicum Mtb* promoter. **(B)** Schematic representation of the dCas9-scRNA complex that includes the dCas9 fused to different activation domains and a gRNA with a 3' extension on its scaffold that serves as anchoring site for the MS2:VPR protein. **(C)** Relative transcriptional activities (RTA) of the tested gRNAs in combination with dCas9:EDLL-MS2:VPR (dCasEV2.1) and a luciferase reporter with the Mtb promoter. **(D)** Comparison of relative transcriptional activities (RTA) obtained using different dCas9:AD (activation domain) versions. The data of bar charts represent the mean average of relative transcriptional activities (RTA) determined as Fluc/Rluc ratios of each sample normalized to Fluc/Rluc ratios of GB1116. The error bars indicate the standard deviations of all biological replicates (*n* = 3). The statistical analyses were performed using unpaired *t*-Test. Asterisks indicate significant differences with CaMV 35s:Luc with a *P-value < 0.05 and **P-value < 0.005.

Unlike the SunTag strategy, scRNA approaches require modified gRNAs with attached RNA aptamers. Therefore, we developed specific GB parts and software-tools for assisting in the assembly of gRNAs for the dCas9-EV2.1 system (Figure 3A). Briefly, single and multiplexed (up to 3X) modified gRNA arrays for transcriptional regulation can be built stepwise using the *Multiple Cas9_gRNA Domesticator tool 1* (*tM9D1*) and *Multiple regulatory Cas9_gRNA Domesticator tool 2* (*tMr9D2*) tools. These tools are equivalent to the above described *tM9D1* and *tM9D2* except that now the input gRNA scaffold contains an RNA aptamer that binds MS2.

Here we tested the functionality of GB4.0-built transcriptional Cas9-based activators by over-activating the *S. lycopersicum Mtb* (Metallothionein-like protein type 2B, Solyc09g010800) promoter in a transient expression experiment in *N. benthamiana*. Given the ability of dCasEV2.1 to activate *DFR* and other inducible promoters, we wanted to investigate here if they could also serve to increase the transcriptional levels conferred by a strong constitutive promoter. The pMtb (cataloged as GB0080) has a strong constitutive activity, reaching approximately four times that of the pNos activity used as reference in GB standard measurements (Vazquez-Vilar et al., 2017). Promoter Relative Transcription Activity (RTA) is estimated in *N. benthamiana* leaves using the Luciferase/Renilla dual reporter system and normalizing the luminescence levels conferred by the test promoter with those produced by the pNos promoter in the same experimental conditions. The resulting normalized value is expressed as relative promoter units (rpu). The RTA for the GB0080 promoter was earlier estimated as 4 +/-1 rpu, about 1/3 of the CaMV 35S promoter (GB0030, RTA = 10 +/−2 rpu). The transcriptional over-activation of the pMtb was first analyzed using the dCas9-EV2.1 complex combined with gRNAs at positions +41, +5, −50, and −402 (relative to the Transcription Start Site, TSS), as represented in Figure 3B. The gRNAs were tested individually or combined in a single T-DNA as depicted. All gRNAs tested in a window of −400 to +5 relative to the TSS conferred strong activation to the promoter, with the gRNA overlapping the TSS showing the maximum activation levels (Figure 3C). The combination of all four gRNAs in a single T-DNA conferred activation levels only slightly higher than those obtained by the gRNA at position +5 acting individually. Most notably, absolute RTA levels obtained with 4X gRNAs reached record RTA levels (72 +/– 8 rpus), corresponding to a 17-fold activation from pMtb basal levels and seven times above CaMV 35S levels used in this experiment as upper limit reference. The high activation conferred by the dCasEV2.1 complex was also confirmed in a separate activation experiment using the same 4X gRNAs but combined with other activation domains in the GB collection (dCas9:VPR-MS2:VPR and dCas9:TV-MS2:VPR). As shown in Figure 3D, the results confirmed earlier observations that the dCasEV2.1 activator complex achieved the highest promoter activation rates.

**Figure 4:**
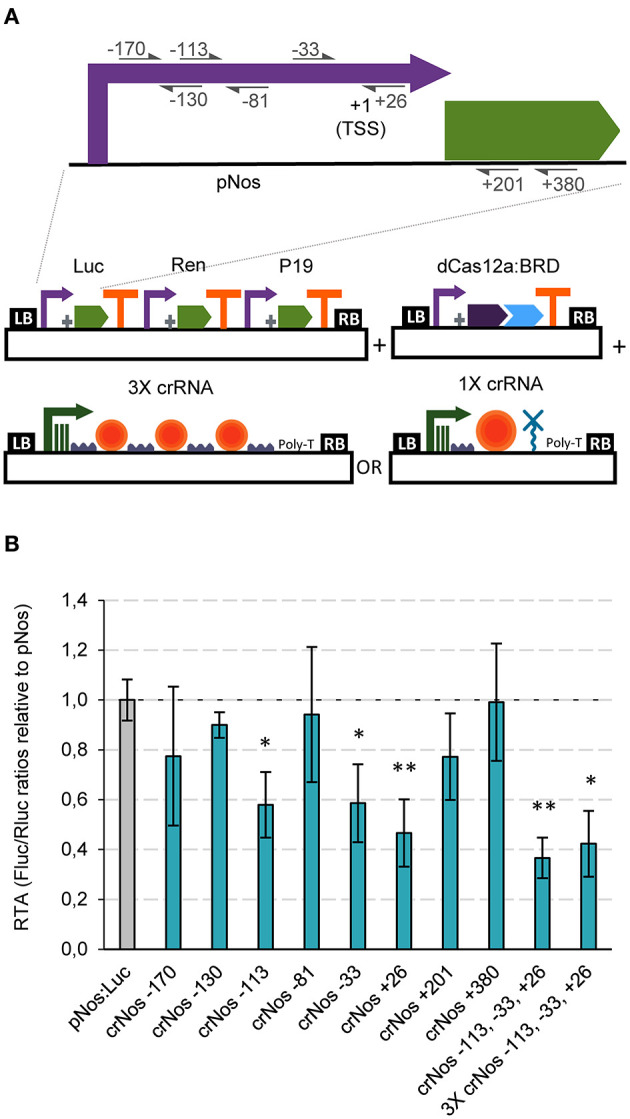
dCas12a-based negative regulation in transient expression. **(A)** Schematic representation of the GB plasmids co-infiltrated for evaluating dCas12a as a tool for negative regulation of the nopaline synthase promoter. **(B)** Relative transcriptional activities (RTA) of the tested gRNAs in combination with the dCas12a:BRD TU and a luciferase reporter with the nos promoter. The data of bar charts represent the mean average of relative transcriptional activities (RTA) determined as Fluc/Rluc ratios of each sample normalized to Fluc/Rluc ratios of GB1116. The error bars indicate the standard deviations of all biological replicates (*n* = 3). The statistical analyses were performed using unpaired *t*-Test. Asterisks indicate significant differences with pNos:Luc with a *P-value < 0.05 and **P-value < 0.005.

Having established strong GB activator tools with dCasEV2.1, we next tested the ability of GB-made dCas12a-based constructs to repress promoter activity. The choice of dCas12a as scaffold for RDs responds to the convenience that activators and repressors operate on different PAMs, therefore facilitating circuit design. Negative regulation is known to result from the activity of the repressor domain, but also from the steric interference of the transcription initiation and elongation complexes, a factor with strong positional dependence. Therefore, to optimize repression strategies we targeted several positions at the pNos promoter, and inside the luciferase coding sequence (positions +201 and +380 in reference to the ATG) (Figure 4A). From those, crRNAs targeting the pNos at −113, −33, and +26 resulted in significant repression rates of 42, 41, and 53%, respectively (Figure 4B). Notably, co-delivery of the three best crRNAs in a single T-DNA, either using single polycistronic TU or multiple TU approaches, resulted in maximum repression rates of 58 and 63%, respectively (Figure 4B).

## Discussion

CRISPR/Cas multiplexing offers unprecedented potential for breeding, but construct assembly is often challenging and cumbersome due to the presence of highly repetitive DNA sequences that prevent chemical synthesis and limit the use of overlapping-based assembly methods. This favors the use of Type IIS-based assembly systems for building multiplex gRNAs. Multiplex assembly systems can be categorized according to their cloning strategy in those that rely on PCR to produce the gRNA units (each unit comprising the processing site, the spacer, the scaffold and the flanking TypeIIS restriction sites for their subsequent cloning) (Vad-Nielsen et al., 2016), and those that incorporate the spacer sequence as overlapping oligonucleotides (McCarty et al., 2019). The latter overcome PCR dependency bypassing the need of sequencing newly generated plasmids (Zuckermann et al., 2018). The individual spacer sequences can be incorporated as annealed oligonucleotides in different plasmids that are later combined to create the polycistronic gRNA (GB strategy) or several pairs of annealed oligos with different overhangs that specify their position in the assembly can be included simultaneously resulting in a one-step assembled polycistronic gRNA (Liao et al., 2019). Despite the second option is faster, it could result in a reduction of the assembly reaction efficiency with the increment in number of the spacer sequences to incorporate in the array. Additionally, one-step assemblies of polycistronic gRNAs limit the promoter selection or the reusability of oligonucleotides for spacers taking part of different polycistronic gRNAs.

With GB4.0 genome edition we want to offer a highly flexible solution for both Cas9 and Cas12a multiplexing, providing all elements required for building any gRNA tandem comprising from 1 to 6 components. In GB we chose to incorporate tRNAs as processable spacers flanking each spacer-scaffold unit in a Cas9 polycistronic gRNA. Previous comparative studies have found little differences in efficiency among the different systems available (Tang et al., 2019). Since the Csy4 system requires the supply in trans of the nuclease, imposing additional cargo to the multigene constructs, we favored the use of the tRNA method, which on the other hand is best suited for modular cloning. In the case of Cas12a, where crRNA processing activity of Cas12a itself is well-established, the GB-proposed multiplexing strategy relied on the direct chemical synthesis of the basic arrays, which are later assembled using regular GB binary-iterative cloning (Bernabé-Orts et al., 2019). To reduce barriers in creating multiplex constructs, we developed an extremely simplify webtool, *CRISPR for Dummies*, which enables minimally trained users to create up to 6X gRNAs ready-to-transform editing construct in a pCAMBIA backbone following a step-by-step guided protocol. We are confident this and other tools will pave the way for many labs to undergo challenging editing projects which would have been technically out of reach without GB4.0 tools in place. All the Cas9 and Cas12a, single and multiple gene editing systems available in the GB4.0 have been functionally validated, and in the case of Cas9 multiplexing, we have performed a stress test to the system by building and testing up to 17 gRNAs in the same construct. The physical linkage of Cas9 and all 17 gRNAs not only ensures multiple targeting but also simplifies the segregation of transgene-free T_1_ plants. The integration of DsRed-positive selection marker greatly simplifies the segregation process (Aliaga-Franco et al., 2019).

Editing efficiency in plants is highly target-dependent, and despite many efforts to develop predictive algorithms (Haeussler et al., 2016), most researchers opt to follow an empirical approach, either performing prior efficiency tests *in vivo* using transient transformation methods (e.g., plant protoplasts), or by using force brute approaches i.e., targeting each gene at several positions. Targeting efficiency is known to be strongly affected by the gRNA sequence itself, with the chromatin accessibility of the target having probably also an influence (Naim et al., 2020). All the gRNAs employed in our stable transformation experiments were selected using the “Rule Set 2 scoring” algorithm (Doench et al., 2016), showing all except one score values above 61. However, our experiments showed that in polycistronic gRNA arrays extra considerations, such as the position of each gRNA in the array, need to be made for targeting efficiency prediction. The editing experiment described in this work, involving 17 gRNAs, >1,000 editing events in a total of 22 T_0_ and 29 T_1_ plants, allowed us to detect influences of the gRNA position in the editing efficiency. Our data shows that all positions are functional, however the last position in the array tends to produce higher efficiency levels, despite the predicted on-target score. A possible explanation for this bias is that the last position is flanked by a single tRNA, whereas the remaining positions need the release of both 5'and 3' flanking tRNAs to produce a functional gRNA. If the nuclear supply of RNase P and RNase Z is a limiting factor, this could explain the observed differences. Alternatively, an increased stability of the 3' end of the RNA due to the presence of the polyA in the last position could also explain this preponderance. Whatever the mechanisms, this bias should be considered in the design of multiplexing constructs, as it might be advisable for instance to locate the most important guides (e.g., those targeting a larger number of orthologs) in the last position. In other occasions, it may be advisable to favor smaller tandems to maximize the number of last-position gRNAs, reaching a balance between multiplexing force and guide efficiency. Despite the observed bias, it becomes clear that multiplex strategies provide unprecedented power to breeding programs, especially in polyploid species. In this work we report the simultaneous biallelic KO of nine *SPL* genes in tobacco in a single generation. Similarly, Stuttmann et al. reported recently the generation of *N. benthamiana* biallelic KOs in eight genes (Stuttmann et al., 2021), in their case using individual Pol III promoters for gRNA expression. Interestingly, besides obtaining full-knock outs, multiplexing has the strength to generate a new type of “targeted” genetic variability that focuses in a specific group of genomic loci, e.g., a large gene family as shown in this work. This custom variability can unveil phenotypes that remained hidden to natural diversity, especially in polyploid species, due to high functional redundancy. In the case of *SPL* genes, the T_1_ plants show strong phenotypes involving flowering time, branching and leaf juvenility, which could not have been generated without a concentrated multiplexing approach.

Finally, the assembly of functional modules to dCas has resulted in a variety of new powerful tools as programmable transcriptional regulators, base editors or prime editing elements among others (Shrestha et al., 2018; Lin et al., 2020; Mishra et al., 2020). We have focused in enriching the GB collection with elements that facilitate programmable transcriptional regulation. Whereas, the successful activation on inducible genes has been earlier reported, here we show that CRISPR/Cas activators can also over-induce a strong promoter, boosting its activity above the level of CaMV 35S promoter. The high expression levels obtained with dCasEV2.1-activated pMtb suggest that this strategy could be exploited to boost yields of recombinant proteins and/or metabolites in molecular farming and/or metabolic engineering approaches. In combination with Cas12a programmable repression in a multiplexing-enabled context, the new tools provide the ability to divert and tune endogenous metabolic pathways, channeling them toward the production of metabolites of interest. Furthermore, the use of different Cas enzymes for positive and negative regulators also facilitates the design of genetic switches. However, in our hands negative regulation is still only partially efficient, and new improvements will be required to achieve stronger repression rates that allow the introduction of Boolean logic approximations for the design of genetic circuits (McCarty et al., 2020).

As in previous versions of the GoldenBraid system, GB4.0 provides a platform from which users can develop their own sub-collections. In the past, other groups have created independently GB extensions for plastids, mitochondria, yeast, gemini viruses, filamentous fungi, mammalian cells or amoebae (Vafaee et al., 2014; Pérez-González et al., 2017; Dahan-Meir et al., 2018; Hernanz-Koers et al., 2018; Sarrion-Perdigones et al., 2019; Kundert et al., 2020). Some extensions have been incorporated to the GB web, such as FungalBraid for filamentous fungi (Vazquez-Vilar et al., 2020), whereas others are internally developed by individual labs. We are confident that the development of multipurpose platforms as GB4.0 is the way to go for turning plant biotechnology into a fast-advancing truly engineering discipline, and this can make a difference in the accessibility of many labs to genome editing and other related technologies.

## Data Availability Statement

The raw data supporting the conclusions of this article will be made available by the authors, without undue reservation.

## Author Contributions

MV-V, SS, JB-O, and DO designed the experiments. MV-V, SS, JB-O, JS-V, BS-S, AR, CP, JQ, and MA conducted the experiments. VG-C developed the software tools. AF-d-C designed the website. MV-V and DO drafted the manuscript. MV-V, AF-d-C, AG, and DO discussed and revised the manuscript. All the authors read and approved the final manuscript.

## Conflict of Interest

JQ was employed by the company Idoasis 2002 S.L. The remaining authors declare that the research was conducted in the absence of any commercial or financial relationships that could be construed as a potential conflict of interest.
