# Supplementary material for: The GB4.0 Platform, an All-In-One Tool for CRISPR/Cas-Based Multiplex Genome Engineering in Plants
Source: Front Plant Sci. 2021 Jul 1;12:689937. doi: 10.3389/fpls.2021.689937 (PMC8284049; doi:10.3389/fpls.2021.689937)
Supplement: Supplementary file 1 [file Data_Sheet_1.docx]

**Supplementary information**

**(E)**

**(D)**

**(C)**

**(B)**

**(A)**


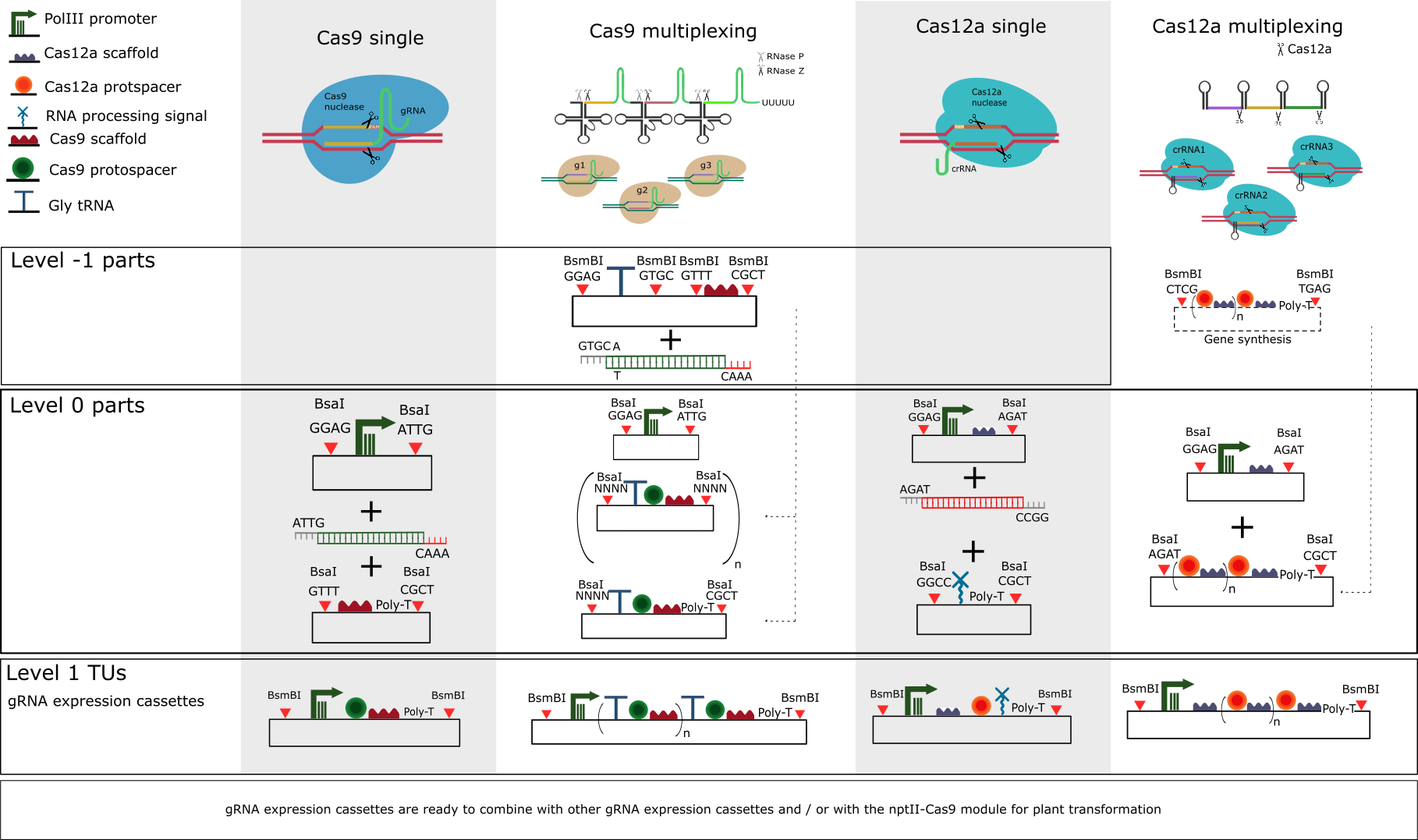


**Supplementary Figure 1. Cas9 and Cas12a single and multiplexing guide RNA expression cassettes cloning strategies with GoldenBraid. (A)** Visual SBOL glyphs for the Level 0 parts involved in the gRNAs expression cassettes assembly. **(B)** Cas9 single gRNAs are assembled as Level 1 constructs with a PolIII promoter, hybridized primers including the protospacer sequence and the corresponding overhangs, and the Cas9 scaffold. **(C)** Cas9 multiplexing gRNAs assembly involves the assembly of Level 0 tRNA-protospacer-scaffold units using hybridized primers including the protospacer sequences and the corresponding overhangs, and the corresponding Level -1 vectors. Level 0 tRNA-protospacer-scaffold units are assembled together and with a PolII promoter to create a Level 1 polycistronic gRNA expression cassette. **(D)** Cas12a single gRNAs are assembled as Level 1 constructs with a PolIII promoter including the Cas12a scaffold, hybridized primers including the protospacer sequences and the corresponding overhangs and a 3’ RNA processing signal. **(E)** Cas12a multiplexing gRNAs assembly involves the synthesis of (protospacer-scaffold)_n_ and the subsequent cloning of the synthetic fragment as a Level 0 part for further assembly with a PolIII promoter including the Cas12a scaffold in Level 1.

**
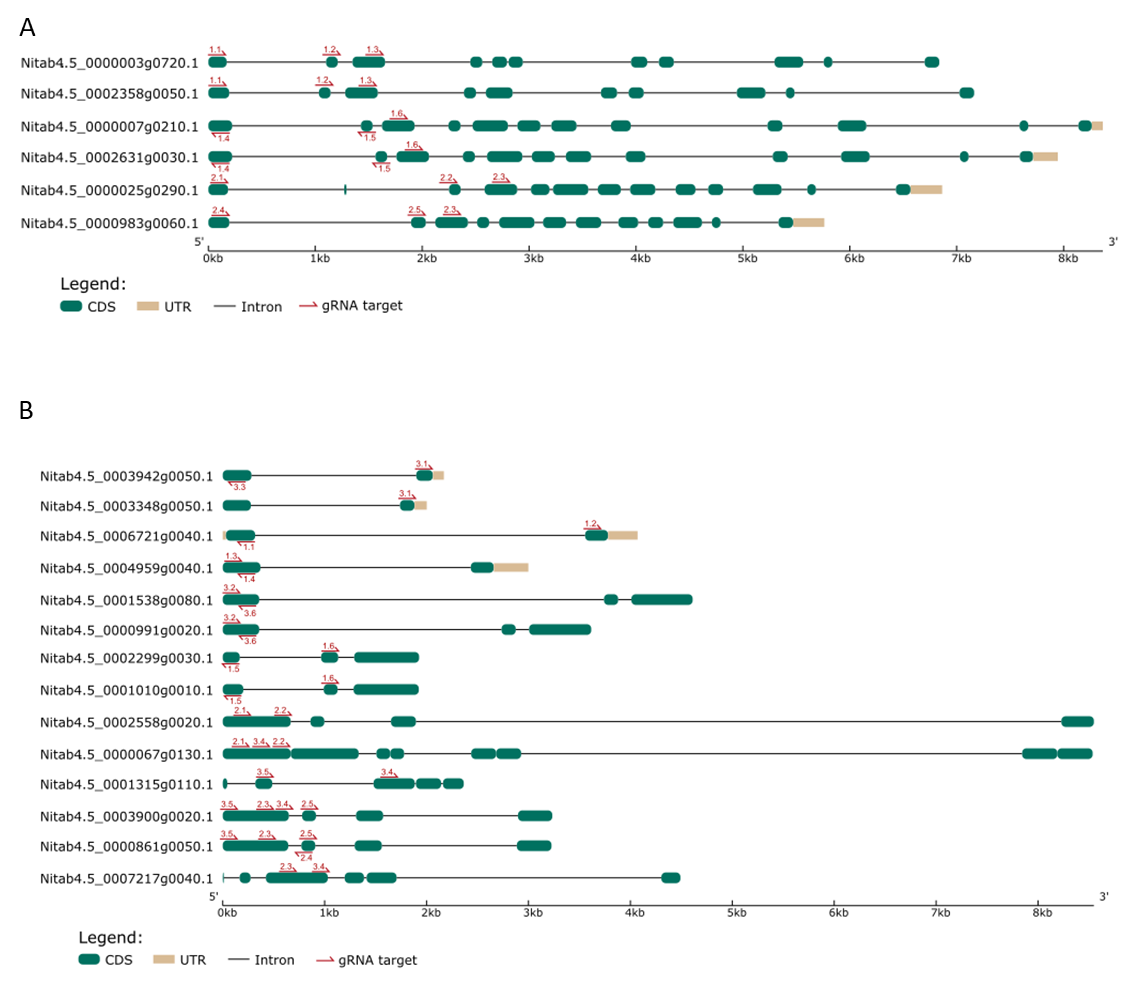
**

**Supplementary Figure 2. Schematic representation of the exon-intron gene structure for the targeted genes.** Exon-intron representation of the targeted SPL genes with red arrows indicating the approximate positions targeted by each gRNA in GB2714.

**Supplementary Table 1. List of GoldenBraid software tools devoted to Cas9 and Cas12a single and polycistronic gRNAs/crRNAs assembly developed in this work.**

| **Tool short name** | **Tool name** | **Purpose** | **Link** |
| --- | --- | --- | --- |
| *tS9D* | *Single Cas9_gRNA domesticator tool* | Protospacer adaptation for Cas9 single gRNAs | <https://gbcloning.upv.es/do/crispr/Single_Cas9_gRNA_Domesticator> |
| *tS9A* | *Single Cas9_gRNA Assembler tool* | Level 1 assembly of Cas9 single gRNAs | https://gbcloning.upv.es/tools/crisprsassembler/ |
| *tS12D* | *Single Cas12a_crRNA domesticator* | Protospacer adaptation for Cas12a single crRNAs | https://gbcloning.upv.es/do/crispr/Single_Cas12a_gRNA_Domesticator |
| *tS12A* | *Single Cas12a_crRNA assembler* | Level 1 assembly of Cas12a single crRNAs | https://gbcloning.upv.es/do/crispr/cas12_single/Single_Cas12a_gRNA_assembler |
| *tM9D1* | *Multiple Cas9_gRNA domesticator tool 1* | Protospacer adaptation for Cas9 polycistronic gRNAs | https://gbcloning.upv.es/do/crispr/multi_cas9_gRNA_domesticator_1 |
| *tM9D2* | *Multiple Cas9_gRNA domesticator tool 2* | Level 0 assembly of Cas9 tRNA-protospacer-scaffold units for polycistronic gRNAs | https://gbcloning.upv.es/do/crispr/multi_cas9_gRNA_domesticator_2/ |
| *tM9A* | *Multiple Cas9_gRNA assembler* | Level 1 assembly of Cas9 polycistronic gRNAs | https://gbcloning.upv.es/tools/cas9multiplexing_eassembler/ |
| *Quick KO* | *CRISPR for Dummies* | Protospacer adaptation + Level 0 assembly of tRNA-protospacer-scaffold units + Level 1 assembly of Cas9 polycistronic gRNAs | https://gbcloning.upv.es/do/crispr/cas9_multiplexing/crispr_for_dummies/ |
| *tM12D* | *Multiple Cas12a_crRNA domesticator* | Protospacer adaptation + Level 0 assembly of Cas12a polycistronic crRNAs | https://gbcloning.upv.es/tools/cas12multiplexing_domestication/ |
| *tM12A* | *Multiple Cas12a_crRNA assembler* | Level 1 assembly of Cas12a polycistronic crRNAs | https://gbcloning.upv.es/tools/cas12multiplexing_assembly/ |
| *tSr9A* | *Single regulatory Cas9_gRNA assembler* | Level 1 assembly of Cas9 single gRNAs for activation | https://gbcloning.upv.es/tools/crisprassemblereg/ |
| *tMr9D2* | *Multiple regulatory Cas9_gRNA Domesticator tool 2* | Level 0 assembly of Cas9 tRNA-protospacer-scaffold with MS2 aptamer units for polycistronic gRNAs for activation | https://gbcloning.upv.es/do/crispr/multi_regulatory_cas9_gRNA_domesticator_2/regulation/ |

**Supplementary Table 2. List of protospacer sequences and targeted genes used in this study.** EDI stands for editing, ACT for activation and REP for repression. *indicates scores estimated with 23 nt genomic sequences corresponding to the crRNA plus additional nucleotides up to 23 nts (CINDEL score is only available for crRNAs of length ≥ 23 nts). gRNA targeted positions were determined as distance of the Cas9/Cas12a cutting site to the ATG for coding sequences and to the TSS for promoter regions. “c” indicates that the gRNA is designed on the coding strand and “nc” that gRNA is designed on the non-coding strand.

| **gRNA name** | **Targeted gene(s) and targeted position** | **Protospacer sequence** | **PAM** | **On-target score^1,2^** | **Cas** | **Purpose** |
| --- | --- | --- | --- | --- | --- | --- |
| gSPL1.1 | Nitab4.5_0006721g0040.1nc260 | CGACGGCTTGAGACTTTGCA | TGG | 64 | Cas9 | EDI |
| gSPL1.2 | Nitab4.5_0006721g0040.1c363 | AATCGAAAAGGAGTTGCAGG | CGG | 70 | Cas9 | EDI |
| gSPL1.3 | Nitab4.5_0004959g0040.1c142 | AAGGGTCAAGTACTTCAATG | AGG | 72 | Cas9 | EDI |
| gSPL1.4 | Nitab4.5_0004959g0040.1nc631 | CGACGGCTTGAGCCTTTGCA | TGG | 60 | Cas9 | EDI |
| gSPL1.5 | Nitab4.5_0002299g0030.1nc135/ Nitab4.5_0001010g0010.1nc123 | GGACCTCACAAACTTTATGG | CGG | 70 | Cas9 | EDI |
| gSPL1.6 | Nitab4.5_0002299g0030.1c274/ Nitab4.5_0001010g0010.1c277 | ATGGACATAACAGGCGTCGA | AGG | 61 | Cas9 | EDI |
| gSPL2.1 | Nitab4.5_0000067g0130.1c419/ Nitab4.5_0002558g0020.1c419 | CGAACAGTTGAAAGCAACAG | TGG | 69 | Cas9 | EDI |
| gSPL2.2 | Nitab4.5_0000067g0130.1c640/ Nitab4.5_0002558g0020.1c640 | TCATCATAGGAGGTGCCGAG | CGG | 74 | Cas9 | EDI |
| gSPL2.3 | Nitab4.5_0000861g0050.1c334/ Nitab4.5_0003900g0020.1c340 / Nitab4.5_0007217g0040.1c466 | TAGAAATTCACCCCCCATGG | AGG | 66 | Cas9 | EDI |
| gSPL2.4 | Nitab4.5_0000861g0050.1nc692 | TGTGATCAGAGAGCCTCCTG | CGG | 66 | Cas9 | EDI |
| gSPL2.5 | Nitab4.5_0003900g0020.1c757 | GGAAACCATCCAGTTCAACT | CGG | 66 | Cas9 | EDI |
| gSPL3.1 | Nitab4.5_0003348g0050.1_c408 / Nitab4.5_0003942g0050.1_c414 | TTAAAGGGGCATCAGTCCAA | TGG | 63 | Cas9 | EDI |
| gSPL3.2 | Nitab4.5_0000991g0020.1_c122 / Nitab4.5_0001538g0080.1_c122 | ATCTACTTTGAAAATGTGGG | TGG | 65 | Cas9 | EDI |
| gSPL3.3 | Nitab4.5_0003348g0050.1_nc212 / Nitab4.5_0003942g0050.1_nc224 | GAAATTCACAGACCTTGTGG | CGG | 73 | Cas9 | EDI |
| gSPL3.4 | Nitab4.5_0001315g0110.1c406/ Nitab4.5_0007217g0040.1c723 / Nitab4.5_0003900g0020.1c598 / Nitab4.5_0000067g0130.1c616 | CAGTCATTCCAAATGCCCAA | AGG | 69 | Cas9 | EDI |
| gSPL3.5 | Nitab4.5_0001315g0110.1c192 | AGATGGAGTGGAATGCAAAG | TGG | 67 | Cas9 | EDI |
| gSPL3.6 | Nitab4.5_0000991g0020.1_nc216 / Nitab4.5_0001538g0080.1_nc216 | CCTTCAACTTGACACCTAGG | TGG | 70 | Cas9 | EDI |
| sgNbALS_c516 | Niben101Scf05032_c516 | GGCGCCACTAATCTCGTCAG | CGG | 72 | Cas9 | EDI |
| sgNbALS_c524 | Niben101Scf05032_c524 | TAATCTCGTCAGCGGCCTCG | CGG | 65 | Cas9 | EDI |
| sgNbALS_c580 | Niben101Scf05032_c580 | TAACCGGTCAAGTGCCACGT | AGG | 70 | Cas9 | EDI |
| sgNbALS_nc583 | Niben101Scf05032_nc583 | TCAGTACCGATCATCCTACG | TGG | 76 | Cas9 | EDI |
| sgNbALS_nc1918 | Niben101Scf05032_nc1918 | GGTAGAACATGTTCCTGATG | AGG | 61 | Cas9 | EDI |
| sgNbALS_c1944 | Niben101Scf05032_c1944 | GTTCTACCTATGATTCCCAG | TGG | 77 | Cas9 | EDI |
| sgNbALS_c1947 | Niben101Scf05032_c1947 | CTACCTATGATTCCCAGTGG | CGG | 68 | Cas9 | EDI |
| crNbALS_c1940 | Niben101Scf05032_c1940 | AAAGCTCCGCCACTGGGAATCAT | TTTG | 19 | Cas12a | EDI |
| crNbALS_c2025 | Niben101Scf05032_c2025 | AGATAATACAAGGTCTAGAACTA | TTTC | 58 | Cas12a | EDI |
| sgNos _c-8 | Nopaline synthase promoter_c-8 | ATTCACTCTCAACTCGATCG | AGG | 65 | Cas9 | EDI |
| crNos:Luc_nc-8 | Nopaline synthase promoter_nc-8 | GCGTCTTCCATTGCCTCGATCGA | TTTG | 57 | Cas12a | EDI |
| sgSlMtb_c-141 | Solyc09g010800_c-141 | ATACGATCACGACACGTGTA | CGG | 63 | Cas9 | EDI |
| sgSlMtb_nc-119 | Solyc09g010800_nc-119 | TATAGTGAAACGAAGGTACA | TGG | 61 | Cas9 | EDI |
| sgSlMtb_nc-112 | Solyc09g010800_nc-112 | GTCTATTTATAGTGAAACGA | AGG | 64 | Cas9 | EDI |
| sgSlMtb_nc-75 | Solyc09g010800_nc-75 | GGTGTAGAAATGAGTGATTG | AGG | 63 | Cas9 | EDI |
| crMtb_c-89 | Solyc09g010800_c-89 | ACTATAAATAGACACTCATGCTT | TTTC | 0.34 | Cas12a | EDI |
| crMtb:Luc_c5 | Firefly Luciferase_c5 | AGACCAATCAATCAATGGAAGAC | TTTC | 0.48 | Cas12a | EDI |
| cr35s_c-62 | CaMV35s_c-62 | GAGAGGACTCCGGTATTTTTACA | TTTG | 0.44 | Cas12a | EDI |
| cr35s_c-41 | CaMV35s_c-41 | CAACAATTACCACAACAAAACAA | TTTA | 0.54 | Cas12a | EDI |
| cr35s:Ren_nc-11 | CaMV35s:Renilla_nc-11 | GAAGTCATTTCGACTAGAATAGT | TTTC | 0.56 | Cas12a | EDI |
| sgCBP_c487 | Niben101Scf02459g00024.1_c487 | GAGGACAAACTACATCCAGG | TGG | 78 | Cas9 | EDI |
| sgFT_nc62 | Niben101Scf01519g10008.1_nc62 | GGCCAATAGATCTTGTAAAA | GGG | 45 | Cas9 | EDI |
| crFT_c83 | Niben101Scf01519g10008.1_c83 | CAAGATCTATTGGCCTAAGAG | TTTA | 0.23 | Cas12a | EDI |
| sgSlMTB_nc41 | Solyc09g010800_nc41 (-155 in reference to ATG) | GTACACGTGTCGTGATCGTA | TGG | 55 | Cas9 | ACT |
| sgSlMTB_c5 | Solyc09g010800_c5  (-191 in reference to ATG) | GATGAAATTAGGATCATGTA | TGG | 52 | Cas9 | ACT |
| sgSlMTB_c-50 | Solyc09g010800_c-50  (-246 in reference to ATG) | GTCTAGAACATACGTACGAA | TGG | 59 | Cas9 | ACT |
| sgSlMTB_nc-402 | Solyc09g010800_nc-402  (-598 in reference to ATG) | GATGTAGCATATGAGATGAT | AGG | 60 | Cas9 | ACT |
| crLuc_nc380 | Firefly Luciferase_nc380 | CAACCCCTTTTTGGAAACGA | TTTG | 0.18 | Cas12a | REP |
| crLuc_nc201 | Firefly Luciferase_nc201 | TATTCAGCCCATATCGTTTC | TTTG | 0.11 | Cas12a | REP |
| crNos_nc26 | Nopaline synthase promoter_nc26 (-9 in reference to ATG) | GCGTCTTCCATTGCCTCGAT | TTTG | 0.24 | Cas12a | REP |
| crNos_nc-33 | Nopaline synthase promoter_nc-33 (-67 in reference to the ATG) | TTGTCAAAAATGCTCCACTG | TTTC | 0.08 | Cas12a | REP |
| crNos_nc-81 | Nopaline synthase promoter_nc-81 (-115 in reference to the ATG) | CTAGCTGATAGTGACCTTAG | TTTG | 0.18 | Cas12a | REP |
| crNos_nc-113 | Nopaline synthase promoter_nc-113 (-147 in reference to the ATG) | TGACGTATGTGCTTAGCTCA | TTTC | 0.13 | Cas12a | REP |
| crNos_nc-130 | Nopaline synthase promoter_nc-130 (-164 in reference to the ATG) | ATGAGCTAAGCACATACGTC | TTTA | 0.33 | Cas12a | REP |
| crNos_nc-170 | Nopaline synthase promoter_nc-170 (-204 in reference to the ATG) | GAACTGACAGAACCGCAACG | TTTG | 0.25 | Cas12a | REP |

^1^ Doench, J. G., Fusi, N., Sullender, M., Hegde, M., Vaimberg, E. W., Donovan, K. F., Smith, I., Tothova, Z., Wilen, C., Orchard, R., et al. (2016). Optimized sgRNA design to maximize activity and minimize off-target effects of CRISPR-Cas9. *Nat Biotechnol* 34:184–191.

^2^Kim, H. K., Song, M., Lee, J., Menon, A. V., Jung, S., Kang, Y.-M., Choi, J. W., Woo, E., Koh, H. C., Nam, J.-W., et al. (2017). In vivo high-throughput profiling of CRISPR–Cpf1 activity. *Nature Methods* 14:153–159.

**Supplementary Table 3. List of plasmids required for the assembly of CRISPR/Cas editing constructs with GoldenBraid.** All these plasmids are available at Addgene (<https://www.addgene.org/>). BA states for Binary Assembler (<https://gbcloning.upv.es/do/bipartite/>). The short names for the rest of the tools can be found in Supplementary Table 1.

| **GB ID** | **Name** | **Purpose** | **Related tool(s)** | **Related publication** |
| --- | --- | --- | --- | --- |
| GB0015 | pDGB_3α1 | Level 1, >1 destination vector | tS9A, tS12A, tM9A, tM12A, BA | Sarrion-Perdigones et al., 2013 |
| GB0017 | pDGB_3α2 | Level 1, >1 destination vector | tS9A, tS12A, tM9A, tM12A, BA | Sarrion-Perdigones et al., 2013 |
| GB0019 | pDGB_3Ω1 | Level >1 destination vector | BA | Sarrion-Perdigones et al., 2013 |
| GB0021 | pDGB_3Ω2 | Level >1 destination vector | BA | Sarrion-Perdigones et al., 2013 |
| GB0307 | pUPD2 | Level 0 destination vector | tM9D2, tM12D | Sarrion-Perdigones et al., 2013 |
| GB1205 | tRNA-scaffold (E1) | 3X, 4X, 5X, 6X Cas9 gRNAs | tM9D2 | Vazquez-Vilar et al., 2016 |
| GB1206 | tRNA-scaffold (E2-E3-E4-En-1) | 3X Cas9 gRNAs | tM9D2 | Vazquez-Vilar et al., 2016 |
| GB1207 | tRNA-scaffold (EnC1) | 2X, 3X, 4X, 5X, 6X Cas9 gRNAs | tM9D2 | Vazquez-Vilar et al., 2016 |
| GB1208 | tRNA-scaffold (E1-E2-E3-E4-En-1) | 2X Cas9 gRNAs | tM9D2 | Vazquez-Vilar et al., 2016 |
| GB2239 | tRNA-scaffold (E2) | 4X, 5X, 6X Cas9 gRNAs | tM9D2 | This work |
| GB2240 | tRNA-scaffold (E3) | 5X, 6X Cas9 gRNAs | tM9D2 | This work |
| GB2241 | tRNA-scaffold (E4) | 6X Cas9 gRNAs | tM9D2 | This work |
| GB2242 | tRNA-scaffold (En-1) | 6X Cas9 gRNAs | tM9D2 | This work |
| GB2243 | tRNA-scaffold (E4-En-1) | 5X Cas9 gRNAs | tM9D2 | This work |
| GB2244 | tRNA-scaffold (E3-E4-En-1) | 4X Cas9 gRNAs | tM9D2 | This work |
| GB2245 | tRNA-scaffold (E3-E4-En-1-EnC1) | 1X Cas9 gRNA | tM9D2 | This work |
| GB1001 | U6-26 (PolIII promoter) | PolIII promoter for single and polycistronic Cas9 gRNAs expression | tS9A, tM9A | Vazquez-Vilar et al., 2016 |
| GB1184 | U6-1 (PolIII promoter) | PolIII promoter for single and polycistronic Cas9 gRNAs expression | tS9A, tM9A | Vazquez-Vilar et al., 2016 |
| GB1204 | U3 (PolIII promoter) | PolIII promoter for single and polycistronic Cas9 gRNAs expression | tS9A, tM9A | Vazquez-Vilar et al., 2016 |
| GB0645 | Cas9 scaffold | *Streptococcus pyogenes* Cas9 scaffold | tS9A | Vazquez-Vilar et al., 2016 |
| GB1443 | U6-26-LbCas12a DR (PolIII promoter + Cas12a direct repeat) | PolIII promoter fused to the *Lachnospiraceae bacterium* Cas12a scaffold for single and polycistronic Cas12a crRNAs expression | tS12A, tM12A | Bernabé‐Orts et al., 2019 |
| GB1444 | HDV (3’ processing signal) | Hammerhead Delta Virus ribozyme for single Cas12a crRNAs | tS12A, tM12A | Bernabé‐Orts et al., 2019 |
| GB0639 | pEGB 35s:Cas9:tNos | TU for the constitutive expression of *Lachnospiraceae bacterium* Cas12a | BA | Vazquez-Vilar et al., 2016 |
| GB2234/GB2235 | pEGB tNos:nptII:PNos-35s:Cas9:tNos-35s:DsRed:tNos | Module for the constitutive expression of nptII, Cas9 and DsRed TUs | BA | This work |
| GB1441 | pEGB 35s:LbCas12a:t35s | TU for the constitutive expression of *Lachnospiraceae bacterium* Cas12a | BA | Bernabé‐Orts et al., 2019 |

Bernabé‐Orts, J. M., Casas‐Rodrigo, I., Minguet, E. G., Landolfi, V., Garcia‐Carpintero, V., Gianoglio, S., et al. (2019). Assessment of Cas12a‐mediated gene editing efficiency in plants. *Plant Biotechnol J* 17, 1971–1984. doi:10.1111/pbi.13113.

Sarrion-Perdigones, A., Vazquez-Vilar, M., Palaci, J., Castelijns, B., Forment, J., Ziarsolo, P., et al. (2013). GoldenBraid 2.0: A Comprehensive DNA Assembly Framework for Plant Synthetic Biology. *PLANT PHYSIOLOGY* 162, 1618–1631. doi:10.1104/pp.113.217661.

Vazquez-Vilar, M., Bernabé-Orts, J. M., Fernandez-Del-Carmen, A., Ziarsolo, P., Blanca, J., Granell, A., et al. (2016). A modular toolbox for gRNA-Cas9 genome engineering in plants based on the GoldenBraid standard. *Plant Methods* 12, 10. doi:10.1186/s13007-016-0101-2.

**Supplementary Table 4. List of plasmids generated during this work.** All sequences and further plasmid’s information can be searched at <https://gbcloning.upv.es/search/features/> with the GB IDs.

| **GB ID** | **Plasmid name** |
| --- | --- |
| GB2714 | pEGB3o1_nptII-Cas9-DsRed-6xsgNtSPL1-5xsgNtSPL2-6xsgNtSPL3 (gSPL1.1-1.6 + gSPL2.1-2.5 + gSPL3.1-3.6) |
| GB2712 | pEGB3a1_nptII-Cas-DsRed-6xsgNtSPL1-5xsgNtSPL2 (gSPL1.1-1.6 + gSPL2.1-2.5) |
| GB2708 | pEGB3a2_6xsgNtSPL3 (gSPL3.1-gSPL3.6) |
| GB2709 | pEGB3o2_6xsgNtSPL1-5xsgNtSPL2 (gSPL1.1-1.6 + gSPL2.1-2.5) |
| GB2706 | pEGB3a1_6xsgNtSPL1 (gSPL1.1-1.6) |
| GB2707 | pEGB3a2_5xsgNtSPL2 (gSPL2.1-2.5) |
| GB2626 | pUPD2_sgNtSPL1.1 [E1] |
| GB2627 | pUPD2_sgNtSPL1.2 [E2] |
| GB2628 | pUPD2_sgNtSPL1.3 [E3] |
| GB2629 | pUPD2_sgNtSPL1.4 [E4] |
| GB2630 | pUPD2_sgNtSPL1.5 [En-1] |
| GB2631 | pUPD2_sgNtSPL1.6 [EnC1] |
| GB2632 | pUPD2_sgNtSPL2.1 [E1] |
| GB2633 | pUPD2_sgNtSPL2.2 [E2] |
| GB2634 | pUPD2_sgNtSPL2.3 [E3] |
| GB2635 | pUPD2_sgNtSPL2.4 [E4-En-1] |
| GB2636 | pUPD2_sgNtSPL2.5 [EnC1] |
| GB2699 | pUPD2_sgNtSPL3.1 [E1] |
| GB2700 | pUPD2_sgNtSPL3.2 [E2] |
| GB2701 | pUPD2_sgNtSPL3.3 [E3] |
| GB2702 | pUPD2_sgNtSPL3.4 [E4] |
| GB2703 | pUPD2_sgNtSPL3.5 [En-1] |
| GB2704 | pUPD2_sgNtSPL3.6 [EnC1] |
| GB2549 | pEGB3α2_U626:pre-tRNA:sgNbALS_c516:SpScaffold |
| GB2550 | pEGB 3α2_U626:pre-tRNA:sgNbALS_c524:SpScaffold |
| GB2551 | pEGB 3α2_U626:pre-tRNA:sgNbALS_c580:SpScaffold |
| GB2552 | pEGB 3α2_U626:pre-tRNA:sgNbALS_nc583:SpScaffold |
| GB3014 | pEGB3α2_U626:pre-tRNA:sgRNA NbALS 1918:SpScaffold |
| GB3015 | pEGB3α2_U626:pre-tRNA:sgRNA NbALS 1944: Scaffold |
| GB3016 | pEGB3α2_U626:pre-tRNA:sgRNA NbALS 1947: Scaffold |
| GB3017 | pEGB3α2_U626:crNbALS_c1940: HDV |
| GB3018 | pEGB3α2_U626:crNbALS_c2025: HDV |
| GB2553 | pEGB3α2_U626:pre-tRNA:sgpNos::Luc_c-8:Scaffold |
| GB2554 | pEGB3α2_U626:LbScaffold:crpNos::Luc_nc-8:HDV |
| GB2555 | pEGB3α2_U626:pre-tRNA:sgSlMtb_c-141:SpScaffold |
| GB2556 | pEGB3α2_U626:pre-tRNA:sgSlMtb _nc-119:SpScaffold |
| GB3583 | pEGB3α2_U626:pre-tRNA:sgSlMtb_nc-112:SpScaffold |
| GB3582 | pEGB3α2_U626:pre-tRNA:sgSlMtb _nc-75:SpScaffold |
| GB2557 | pEGB3α2_U626:LbScaffold:crSlMtb_c-89:HDV |
| GB2558 | pEGB3α2_U626:LbScaffold:crSlMtb::Luc_c5:HDV |
| GB2559 | pEGB3α2_U626:LbScaffold:cr35S_c-62:HDV |
| GB3030 | pEGB3α2_U626:LbScaffold:cr35S_c-41: HDV |
| GB2561 | pEGB3α2_U626:LbScaffold:cr35S::Ren_nc-11: HDV |
| GB1774 | pEGB3α2_U626:sgNbCBP_c487:SpScaffold |
| GB1773 | pEGB3α2_U626:sgNbFT_nc62:SpScaffold |
| GB3033 | pEGB3α2_U626:LbScaffold:crNbFT_c83: HDV |
| GB2541 | pUPD2_sgNbALS_c516 [E1-E2-E3-E4-En-1-EnC1] |
| GB2542 | pUPD2_ sgNbALS_c524 [E1-E2-E3-E4-En-1-EnC1] |
| GB2543 | pUPD2_ sgNbALS_c580 [E1-E2-E3-E4-En-1-EnC1] |
| GB2544 | pUPD2_ sgNbALS_nc583 [E1-E2-E3-E4-En-1-EnC1] |
| GB3002 | pUPD2_ sgNbALS_nc1918 [E1-E2-E3-E4-En-1-EnC1] |
| GB3003 | pUPD2_ sgNbALS_c1944 [E1-E2-E3-E4-En-1-EnC1] |
| GB3004 | pUPD2_ sgNbALS_c1947 [E1-E2-E3-E4-En-1-EnC1] |
| GB2545 | pUPD2_sgNos_c-8 [E1-E2-E3-E4-En-1-EnC1] |
| GB2546 | pUPD2_sgSlMtb_c-141 [E1-E2-E3-E4-En-1-EnC1] |
| GB2547 | pUPD2_sgSlMtb_nc-119 [E1-E2-E3-E4-En-1-EnC1] |
| GB2548 | pUPD2_sgSlMtb_nc-112 [E1-E2-E3-E4-En-1-EnC1] |
| GB3581 | pUPD2_sgSlMtb_nc-75 [E1-E2-E3-E4-En-1-EnC1] |
| GB1119 | pEGB 35s:Luc:TNos-SF-35s:Renilla:TNos-35s:P19:TNos-SF |
| GB1399 | pEGB3alpha2 MTB:luc:Tnos-SF-35S:Ren:Tnos-35s:P19:Tnos-SF |
| GB1830 | 35s:Ms2:VPR:tNos |
| GB2047 | 35s:dCas9:TV:tNos |
| GB1190 | 35s:dCas9:EDLL:tNos |
| GB1826 | 35s:dCas9:VPR:tNos |
| GB2045 | 3alpha1:U6-26:sgSlMtb_nc41_scf F6x2 |
| GB1801 | 3alpha1:U6-26:sgSlMtb_c5_scf F6x2 |
| GB2044 | 3alpha2:U6-26:sgSlMtb_c-50_scf F6x2 |
| GB1859 | 3alpha1:U6-26:sgSlMtb_nc-402_scf F6x2 |
| GB2070 | pEGB3alpha1_U6-26:sgSlMtb_nc-402:F6x2_U6-26:sgSlMtb_c-50:F6x2_U6-26:sgSlMtb_c5:F6x2_U6-26:sgSlMtb_nc41:F6x2 |
| GB1116 | pEGB pNos:Luciferase:TNos-SF-35S:Renilla:TNos-35S:P19:TNos-SF |
| GB1668 | pEGB 3alpha1 35S:dLbCas12a:BRD:T35S |
| GB1805 | pEGB3alpha2_ U626:LbScaffold:crLuc_nc380:HDV |
| GB1806 | pEGB3alpha2_ U626:LbScaffold:crLuc_nc201:HDV |
| GB1807 | pEGB3alpha2_ U626:LbScaffold:crpNos::Luc_nc26:HDV |
| GB1808 | pEGB3alpha2_ U626:LbScaffold:crpNos_nc-33:HDV |
| GB1809 | pEGB3alpha2_ U626:LbScaffold:crpNos_nc-81:HDV |
| GB1811 | pEGB3alpha2_ U626:LbScaffold:crpNos_nc-113:HDV |
| GB1812 | pEGB3alpha2_ U626:LbScaffold:crpNos_nc-130:HDV |
| GB1813 | pEGB3alpha2_ U626:LbScaffold:crpNos_nc-170:HDV |
| GB1879 | U6-26::LbScaffold:crNos_nc-113:LbScaffold:crNos-33:LbScaffold:crNos_nc26:LbDR |

**Supplementary Table 5. List of primers used for amplification of the targeted sites.**

| **Primer Name** | **Sequence (5’ 🡪 3’)** |
| --- | --- |
| MV19OCT01 SPL2299_g1.5_F3 | GTGTGATCGATGGAATCATCA |
| MV19SEP12 SPL2299_g1.5_R2 | ATCTTCCTAACTATGTGGCG |
| MV19AGO03 SPL1010_g1.5_F | GTGTGATCGATGGAATCATCC |
| MV19AGO04 SPL1010_g1.5_R | GTTTCCAGCCTTTATGAGAAC |
| MV19AGO05 SPL2299_g1.6_F | TGCTCTTGAGTCCTGTCC |
| MV19AGO06 SPL2299_g1.6_R | GTTGACTGCTAAATGACAGAAG |
| MV19AGO07 SPL1010_g1.6_F | ATGATGTGTGATTACTTAGCAC |
| MV19AGO08 SPL1010_g1.6_R | GTTGACTGCTAAATGACAGAAC |
| MV19AGO09 SPL0067_g2.1&2.2_F | GAGCTCAATATCAGCTTCTACC |
| MV19AGO10 SPL0067_g2.1&2.2_R | CTATACATCCAGAAGAACAAGTTGTT |
| MV19AGO11 SPL2558_g2.1&2.2_F | GAGAACTTGATCGCAGATCG |
| MV19AGO12 SPL2558_g2.1&2.2_R | GCTATACATCCAGAAGAACACG |
| MV19AGO13 SPL0861_g2.3&g2.4_F | GATTCGAACTTTCCTGTTGTTTC |
| MV19AGO14 SPL0861_g2.3&g2.4_R | CTTTAGCTGATGAAAGATCAAGGT |
| MV19AGO15 SPL3900_g2.3&g2.4_F | GTGTTCTTGCTCTTTGATTCA |
| MV19AGO16 SPL3900_g2.3&g2.4_R | CTTTAGCTGATGAAAGATCATGGC |
| MV19AGO17 SPL0861_g3.4&g2.5_F | GGCTCTGTTGAACCACTTG |
| MV19AGO18 SPL0861_g3.4&g2.5_R | CTGTCCATGTTAACCTATTCTC |
| MV19AGO19 SPL3900_g3.4&g2.5_F | GACATTTGAGACCATCGGC |
| MV19AGO20 SPL3900_g3.4&g2.5_R | GGTTGTTTAACCAGCAAAGAAAC |
| MV19AGO21 SPL7217_g3.5,g2.3&g3.4_F | GGAGATGCATTTTGTGGC |
| MV19AGO22 SPL7217_g3.5,g2.3&g3.4_R | CCAGTTGGGTGTGGTTGAGA |
| MV19AGO61 SPL1315_g3.5_F2 | CAACAGCTCTGTTTGATAAAGATCC |
| MV19AGO62 SPL1315_g3.5_R2 | CAAGTTCTCCATCTTCTTCAAC |
| MV19AGO25 SPL1315_g3.4_F | GGTGCTAAGATTTCATCCTTCG |
| MV19AGO26 SPL1315_g3.4_R | AACATGGATTCCTTGGCATC |
| MV19AGO27 SPL4959_g1.3&g1.4_F | CTTAGCCTATATCTTCCTATAGC |
| MV19AGO28 SPL4959_g1.3&g1.4_R | GTATTTTAGTAACTTAAAAATGTGCTG |
| MV19AGO29 SPL6721_g1.3&g1.1_F | CACTTAGCCTATATCTTCCTAAC |
| MV19AGO30 SPL6721_g1.3&g1.4_R | CAACATGCTAAAGAATGCATC |
| MV19AGO31 SPL6721_g1.2_F | TTCATACACACCTAACAACATGAC |
| MV19AGO32 SPL6721_g1.2_R | CAAATTTCGTTATGTGGGATTACG |
| MV19AGO33 SPL0991_g3.2&g3.6_F | GTATACATGACACTGTGGCTA |
| MV19AGO34 SPL0991_g3.2&g3.6_R | GTGAAATTTCTCTACTCTGTCAGG |
| MV19AGO35 SPL1538_g3.2&g3.6_F | GAACTGGGTTCAGTTTCTTCTC |
| MV19AGO36 SPL1538_g3.2&g3.6_R | GTTTTGTATCCGCGTATCGACA |
| MV19AGO37 SPL3348_g3.3_F | GAGGAAGACGAAGATGTTGTA |
| MV19AGO38 SPL3348_g3.3_R | CATTTTAAGACAACACAAGCACT |
| MV19AGO39 SPL3942_g3.3_F | GAGGAAGACGAAGATGTTGTG |
| MV19AGO40 SPL3942_g3.3_R | GAATTTAACTTTTGTACACTGACC |
| MV19AGO41 SPL3348_g3.1_F | GCAAGGATGTTGTAACTTGGTTC |
| MV19AGO42 SPL3348_g3.1_R | ACAGACAAAGTTAATGACACACA |
| MV19AGO43 SPL3942_g3.1_F | GCAAGGATGTTGTATCTTGGTTG |
| MV19AGO44 SPL3942_g3.1_R | ACAGACAAAGTTAATGACTCACT |
| JS19ENE01_ALS_F/seq | CAACGTCTTTGCGTACCCAGG |
| JS19ENE02_ALS_R | CCCCAAATGCGAGCAACAAATC |
| JS19ENE03_Nos_F/seq | TTGAAGGAGCCACTGAGCC |
| JS19ENE04_Nos_R | GGCTGCGAAATGCCCATACTG |
| JS19ENE05_Mtb_F/seq | AGTCGCGGTCGATAGAGAATG |
| JS19ENE06_Mtb_R | ACATCGACTGAAATCCCTGG |
| JS19ENE07_35s_F/seq | CGAGGAGCATCGTGGAAAAAG |
| JS19ENE08_35s_R | CAGGCCATTCATCCCATGATTC |
| JO16DIC16_FT_F | CTAGAAAACCTATGGCTATAAGGG |
| JO16DIC17_FT_R | GTTCTCGAGAGGTATAATATAGGC |
| JO16DIC18_FTseq | CACAAGCACGCATAGAAC |
| JO17AB21_CBP_F | TGTCTAGACTGGTGCATTACTTC |
| JO17AB21_CBP_R/seq | GTTGCCAAAAGGATCACTCAAAT |

**Supplementary Table 6. Mutations of the SPL T_1_ plants described in this work.**

All raw chromatograms and Synthego analyses for the T_1_ *SPL* edited lines generated in this work are available at <https://doi.org/10.5281/zenodo.4777581>. As example, Synthego results for plant SPL15-1 are shown here.

**Plant SPL2-1**

Nitab_0002299g0030.1

| *gSPL1.6 seq:* | ATGGACATAACAGGCGT\|CGAAGGAAACCTCAGCC | Mut | Genotype |
| --- | --- | --- | --- |
| Allele 1: | ATGGACATAACAGGCGT\|ACGAAGGAAACCTCAGCC | +1 | BA |
| Allele 2: | ATGGA------------\|-----------TCAGCC | -23 |  |

Nitab_0001010g0010.1

| *gSPL1.6 seq:* | ATGGACATAACAGGCGT\|CGAAGGAAACCTCAGCC | Mut | Genotype |
| --- | --- | --- | --- |
| Allele 1: | ATGGACATAACAGGCGT\|TCGAAGGAAACCTCAGCC | +1 | HM |
| Allele 2: | ATGGACATAACAGGCGT\|TCGAAGGAAACCTCAGCC | +1 |  |

Nitab_0000861g0050.1

| *gSPL2.4 seq:* | TGTGATCAGAGAGCCTC\|CTGCGGCAGCTTCTCT | Mut | Genotype |
| --- | --- | --- | --- |
| Allele 1: | TGTGATCAGAGAGCCT-\|CTGCGGCAGCTTCTCT | -1 | HM |
| Allele 2: | TGTGATCAGAGAGCCT-\|CTGCGGCAGCTTCTCT | -1 |  |
| *gSPL3.5 seq:* | AGATGGAGTGGAATGCA\|AAGTGGGACTGGGGAAA | Mut | Genotype |
| Allele 1: | AGATGGAGTGGAATGCA\|AAGTGGGACTGGGGAAA | WT | HT |
| Allele 2: | AGATGGAGTGGAATGCA\|AAAGTGGGACTGGGGAAA | +1 |  |

**Plant SPL2-3**

Nitab_0003942g0050.1

| *gSPL3.1 seq:* | TTAAAGGGGCATCAGTC\|CAATGGCAGAGAAATC | Mut | Genotype |
| --- | --- | --- | --- |
| Allele 1: | TTAAAGGGGCATCAGTC\|CCAATGGCAGAGAAATC | +1 | HM |
| Allele 2: | TTAAAGGGGCATCAGTC\|CCAATGGCAGAGAAATC | +1 |  |

Nitab_0001538g0080.1

| *gSPL3.6 seq:* | CCTTCAACTTGACACCT\|AGGTGGCTGACCACCCT | Mut | Genotype |
| --- | --- | --- | --- |
| Allele 1: | CCTTCAACTTGA----T\|AGGTGGCTGACCACCCT | -4 | HM |
| Allele 2: | CCTTCAACTTGA----T\|AGGTGGCTGACCACCCT | -4 |  |

Nitab_0002299g0030.1

| *gSPL1.6 seq:* | ATGGACATAACAGGCGT\|CGAAGGAAACCTCAGCC | Mut | Genotype |
| --- | --- | --- | --- |
| Allele 1: | ATGGACATAACAGGCGT\|ACGAAGGAAACCTCAGCC | +1 | HM |
| Allele 2: | ATGGACATAACAGGCGT\|ACGAAGGAAACCTCAGCC | +1 |  |

Nitab_0001010g0010.1

| *gSPL1.6 seq:* | ATGGACATAACAGGCGT\|CGAAGGAAACCTCAGCC | Mut | Genotype |
| --- | --- | --- | --- |
| Allele 1: | ATGGACATAACAGGCGT\|TCGAAGGAAACCTCAGCC | +1 | HM |
| Allele 2: | ATGGACATAACAGGCGT\|TCGAAGGAAACCTCAGCC | +1 |  |

Nitab_0000067g0130.1

| *gSPL2.1 seq:* | CGAACAGTTGAAAGCAA\|CAGTGGTGGAAGCAGT | Mut | Genotype |
| --- | --- | --- | --- |
| Allele 1: | CGAACAGTTGAAAGCAA\|ACAGTGGTGGAAGCAGT | +1 | HM |
| Allele 2: | CGAACAGTTGAAAGCAA\|ACAGTGGTGGAAGCAGT | +1 |  |

Nitab_0000861g0050.1

| *gSPL3.5 seq:* | AGATGGAGTGGAATGCA\|AAGTGGGACTGGGGAAA | Mut | Genotype |
| --- | --- | --- | --- |
| Allele 1: | AGATGGAGTGGAATGCA\|AAAGTGGGACTGGGGAAA | +1 | HM |
| Allele 2: | AGATGGAGTGGAATGCA\|AAAGTGGGACTGGGGAAA | +1 |  |

**Plant SPL2-5**

Nitab_0003942g0050.1

| *gSPL3.1 seq:* | TTAAAGGGGCATCAGTC\|CAATGGCAGAGAAATC | Mut | Genotype |
| --- | --- | --- | --- |
| Allele 1: | TTAAAGGGGCATCAGTC\|CAATGGCAGAGAAATC | WT | HT |
| Allele 2: | TTAAAGGGGCATCAGTC\|CCAATGGCAGAGAAATC | +1 |  |

Nitab_0001538g0080.1

| *gSPL3.6 seq:* | CCTTCAACTTGACACCT\|AGGTGGCTGACCACCCT | Mut | Genotype |
| --- | --- | --- | --- |
| Allele 1: | CCTTCAACTTGA----T\|AGGTGGCTGACCACCCT | -4 | HM |
| Allele 2: | CCTTCAACTTGA----T\|AGGTGGCTGACCACCCT | -4 |  |

Nitab_0002299g0030.1

| *gSPL1.6 seq:* | ATGGACATAACAGGCGT\|CGAAGGAAACCTCAGCC | Mut | Genotype |
| --- | --- | --- | --- |
| Allele 1: | ATGGACATAACAGGCGT\|ACGAAGGAAACCTCAGCC | +1 | HM |
| Allele 2: | ATGGACATAACAGGCGT\|ACGAAGGAAACCTCAGCC | +1 |  |

Nitab_0001010g0010.1

| *gSPL1.6 seq:* | ATGGACATAACAGGCGT\|CGAAGGAAACCTCAGCC | Mut | Genotype |
| --- | --- | --- | --- |
| Allele 1: | ATGGACATAACAGGCGT\|TCGAAGGAAACCTCAGCC | +1 | HM |
| Allele 2: | ATGGACATAACAGGCGT\|TCGAAGGAAACCTCAGCC | +1 |  |

Nitab_0000067g0130.1

| *gSPL2.1 seq:* | CGAACAGTTGAAAGCAA\|CAGTGGTGGAAGCAGT | Mut | Genotype |
| --- | --- | --- | --- |
| Allele 1: | CGAACAGTTGAAAGCAA\|CAGTGGTGGAAGCAGT | WT | HT |
| Allele 2: | CGAACAGTTGAAAGCAA\|ACAGTGGTGGAAGCAGT | +1 |  |

Nitab_0000861g0050.1

| *gSPL2.4 seq:* | TGTGATCAGAGAGCCTC\|CTGCGGCAGCTTCTCTTCTTTT | Mut | Genotype |
| --- | --- | --- | --- |
| Allele 1: | TGTGATCAGAGAGCCT-\|CTGCGGCAGCTTCTCTTCTTTT | -1 | HM |
| Allele 2: | TGTGATCAGAGAGCCT-\|CTGCGGCAGCTTCTCTTCTTTT | -1 |  |
| *gSPL3.5 seq:* | AGATGGAGTGGAATGCA\|AAGTGGGACTGGGGAAA | Mut | Genotype |
| Allele 1: | AGATGGAGTGGAATGCA\|AAGTGGGACTGGGGAAA | WT | HT |
| Allele 2: | AGATGGAGTGGAATGCA\|AAAGTGGGACTGGGGAAA | +1 |  |

**Plant SPL10-3**

Nitab_0003942g0050.1

| *gSPL3.1 seq:* | TTAAAGGGGCATCAGTC\|CAATGGCAGAGAAATC | Mut | Genotype |
| --- | --- | --- | --- |
| Allele 1: | TTAAAGGGGCATCAGTC\|CAATGGCAGAGAAATC | WT | HT |
| Allele 2: | TTAAAGGGGCATCAGTC\|TCAATGGCAGAGAAATC | +1 |  |
| gSPL3.3 seq: | GAAATTCACAGACCTTG\|TGGCGGCGATGGTATGTC | Mut | Genotype |
| Allele 1: | GAAATTCACAGACCTTG\|TGGCGGCGATGGTATGTC | WT | HT |
| Allele 2: | GAAATTCACAGACCTT-\|TGGCGGCGATGGTATGTC | -1 |  |

Nitab_0003348g0050.1

| *gSPL3.1 seq:* | TTAAAGGGGCATCAGTC\|CAATGGCAGAGAAATC | Mut | Genotype |
| --- | --- | --- | --- |
| Allele 1: | TTAAAGGGGCATCAGTC\|TCAATGGCAGAGAAATC | +1 | HM |
| Allele 2: | TTAAAGGGGCATCAGTC\|TCAATGGCAGAGAAATC | +1 |  |

Nitab_0001538g0080.1

| *gSPL3.6 seq:* | CCTTCAACTTGACACCT\|AGGTGGCTGACCACCCT | Mut | Genotype |
| --- | --- | --- | --- |
| Allele 1: | CCTTCAACTTGACAC-A\|AGGTGGCTGACCACCCT | -2/+1 | BA |
| Allele 2: | CCTTCAACTTGACAC--\|AGGTGGCTGACCACCCT | -2 |  |
| *gSPL3.2 seq:* | ATCTACTTTGAAAATG\|TGGTGGGTCATCGCCGGT | Mut | Genotype |
| Allele 1: | ATCTACTTTGAAAATG\|TGGTGGGTCATCGCCGGT | WT | HT |
| Allele 2: | ATCTACTTTG------\|-GGTGGGTCATCGCCGGT | -7 |  |

Nitab_0000991g0020.1

| *gSPL3.6 seq:* | CCTTCAACTTGACACCT\|AGGTGGCTGACCACCCTGT | Mut | Genotype |
| --- | --- | --- | --- |
| Allele 1: | CCTTCAACTTGACACCT\|AAGGTGGCTGACCACCCTGT | +1 | HM |
| Allele 2: | CCTTCAACTTGACACCT\|AAGGTGGCTGACCACCCTGT | +1 |  |

Nitab_0002299g0030.1

| *gSPL1.6 seq:* | ATGGACATAACAGGCGT\|CGAAGGAAACCTCAGCC | Mut | Genotype |
| --- | --- | --- | --- |
| Allele 1: | ATGGACATAACAGGCGT\|TCGAAGGAAACCTCAGCC | +1 | BA |
| Allele 2: | ATGGACATAACAGGCGT\|ACGAAGGAAACCTCAGCC | +1 |  |

Nitab_0001010g0010.1

| *gSPL1.6 seq:* | ATGGACATAACAGGCGT\|CGAAGGAAACCTCAGCC | Mut | Genotype |
| --- | --- | --- | --- |
| Allele 1: | ATGGACATAACAGGCGT\|TCGAAGGAAACCTCAGCC | +1 | HM |
| Allele 2: | ATGGACATAACAGGCGT\|TCGAAGGAAACCTCAGCC | +1 |  |

Nitab_0001315g0110.1

| *gSPL3.5 seq:* | AGATGGAGTGGAATGCA\|AAGTGGGACTGGGGAAACCA | Mut | Genotype |
| --- | --- | --- | --- |
| Allele 1: | AGATGGAGTGGAATGCA\|AAAGTGGGACTGGGGAAACCA | +1 | HM |
| Allele 2: | AGATGGAGTGGAATGCA\|AAAGTGGGACTGGGGAAACCA | +1 |  |

Nitab_0003900g0020.1

| *gSPL2.5 seq:* | GGAAACCATCCAGTTCA\|ACTCGGCAAGGCTTTCTTCAT | Mut | Genotype |
| --- | --- | --- | --- |
| Allele 1: | GGAAACCATCCAGTT--\|ACTCGGCAAGGCTTTCTTCAT | -2 | BA |
| Allele 2: | GGAAACCATCCAG---A\|ACTCGGCAAGGCTTTCTTCAT | -3 |  |

Nitab_0000861g0050.1

| *gSPL2.4 seq:* | TGTGATCAGAGAGCCTC\|CTGCGGCAGCTTCTCTTCTTT | Mut | Genotype |
| --- | --- | --- | --- |
| Allele 1: | TGTGATCAGAGAG---C\|CTGCGGCAGCTTCTCTTCTTT | -3 | HM |
| Allele 2: | TGTGATCAGAGAG---C\|CTGCGGCAGCTTCTCTTCTTT | -3 |  |

Nitab_0007217g0040.1

| *gSPL2.3 seq:* | TAGAAATTCACCCCCCA\|TGGAGGCTTTGGTAGGCT | Mut | Genotype |
| --- | --- | --- | --- |
| Allele 1: | TAGAAATT---------\|TGGAGGCTTTGGTAGGCT | -9 | HM |
| Allele 2: | TAGAAATT---------\|TGGAGGCTTTGGTAGGCT | -9 |  |

**Plant SPL10-5**

Nitab_0003942g0050.1

| *gSPL3.1 seq:* | TTAAAGGGGCATCAGTC\|CAATGGCAGAGAAATC | Mut | Genotype |
| --- | --- | --- | --- |
| Allele 1: | TTAAAGGGGCATCAGTC\|TCAATGGCAGAGAAATC | +1 | HM |
| Allele 2: | TTAAAGGGGCATCAGTC\|TCAATGGCAGAGAAATC | +1 |  |
| gSPL3.3 seq: | GAAATTCACAGACCTTG\|TGGCGGCGATGGTATGTC | Mut | Genotype |
| Allele 1: | GAAATTCACAGACCTT-\|TGGCGGCGATGGTATGTC | -1 | HM |
| Allele 2: | GAAATTCACAGACCTT-\|TGGCGGCGATGGTATGTC | -1 |  |

Nitab_0001538g0080.1

| *gSPL3.6 seq:* | CCTTCAACTTGACACCT\|AGGTGGCTGACCACCCT | Mut | Genotype |
| --- | --- | --- | --- |
| Allele 1: | CCTTCAACTTGACAC-A\|AGGTGGCTGACCACCCT | -2/+1 | HM |
| Allele 2: | CCTTCAACTTGACAC-A\|AGGTGGCTGACCACCCT | -2/+1 |  |

Nitab_0000991g0020.1

| *gSPL3.6 seq:* | CCTTCAACTTGACACCT\|AGGTGGCTGACCACCCTGT | Mut | Genotype |
| --- | --- | --- | --- |
| Allele 1: | CCTTCAACTTGACACCT\|AGGTGGCTGACCACCCTGT | WT | HT |
| Allele 2: | CCTTCAACTTGACACCT\|TAGGTGGCTGACCACCCTGT | +1 |  |

Nitab_0002299g0030.1

| *gSPL1.6 seq:* | ATGGACATAACAGGCGT\|CGAAGGAAACCTCAGCC | Mut | Genotype |
| --- | --- | --- | --- |
| Allele 1: | ATGGACATAACAGGCGT\|TCGAAGGAAACCTCAGCC | +1 | HM |
| Allele 2: | ATGGACATAACAGGCGT\|TCGAAGGAAACCTCAGCC | +1 |  |

Nitab_0001010g0010.1

| *gSPL1.6 seq:* | ATGGACATAACAGGCGT\|CGAAGGAAACCTCAGCC | Mut | Genotype |
| --- | --- | --- | --- |
| Allele 1: | ATGGACATAACAGGCGT\|TCGAAGGAAACCTCAGCC | +1 | BA |
| Allele 2: | ATGGACATAACAGGCGT\|CCGAAGGAAACCTCAGCC | +1 |  |

Nitab_0003900g0020.1

| *gSPL2.5 seq:* | GGAAACCATCCAGTTCA\|ACTCGGCAAGGCTTTCTTCAT | Mut | Genotype |
| --- | --- | --- | --- |
| Allele 1: | GGAAACCATCCAG---A\|ACTCGGCAAGGCTTTCTTCAT | -3 | HM |
| Allele 2: | GGAAACCATCCAG---A\|ACTCGGCAAGGCTTTCTTCAT | -3 |  |

Nitab_0000861g0050.1

| *gSPL2.4rc & 2.5 seq:* | CCGCAGGAGGCTCTCTGATCACAATGCACGACGCCGCAA ACCACAGCAGGAAACCATCCAGTTCAACTCGGCAAGGCT | Mut | Genotype |
| --- | --- | --- | --- |
| Allele 1: | CCGCAG--------------------------------- --------------------------ACTCGGCAAGGCT | -59 | HM |
| Allele 2: | CCGCAG--------------------------------- --------------------------ACTCGGCAAGGCT | -59 |  |

Nitab_0007217g0040.1

| *gSPL2.3 seq:* | TAGAAATTCACCCCCCA\|TGGAGGCTTTGGTAGGCT | Mut | Genotype |
| --- | --- | --- | --- |
| Allele 1: | TAGAAATTCACCCCCCA\|TGGAGGCTTTGGTAGGCT | WT | HT |
| Allele 2: | TAGAAATT---------\|TGGAGGCTTTGGTAGGCT | -9 |  |

**Plant SPL11-3**

Nitab_0004959g0040.1

| *gSPL1.3 seq:* | AAGGGTCAAGTACTTCA\|ATGAGGTGTTGCCAAGCTGA | Mut | Genotype |
| --- | --- | --- | --- |
| Allele 1: | AAGGGTCAAGTACTTCA\|ATGAGGTGTTGCCAAGCTGA | WT | HT |
| Allele 2: | AAGGGTCAAGTACTTCA\|AATGAGGTGTTGCCAAGCTGA | +1 |  |

Nitab_0002299g0030.1

| *gSPL1.6 seq:* | ATGGACATAACAGGCGT\|CGAAGGAAACCTCAGCC | Mut | Genotype |
| --- | --- | --- | --- |
| Allele 1: | ATGGACATAACAGGCGT\|TCGAAGGAAACCTCAGCC | +1 | BA |
| Allele 2: | ATGGACATAACAGGCGT\|ACGAAGGAAACCTCAGCC | +1 |  |

Nitab_0001010g0010.1

| *gSPL1.6 seq:* | ATGGACATAACAGGCGT\|CGAAGGAAACCTCAGCC | Mut | Genotype |
| --- | --- | --- | --- |
| Allele 1: | ATGGACATAACAGGCGT\|ACGAAGGAAACCTCAGCC | +1 | HM |
| Allele 2: | ATGGACATAACAGGCGT\|ACGAAGGAAACCTCAGCC | +1 |  |

Nitab_0000861g0050.1

| *gSPL2.4 seq:* | TGTGATCAGAGAGCCTC\|CTGCGGCAGCTTCTCTTCTTT | Mut | Genotype |
| --- | --- | --- | --- |
| Allele 1: | TGTGATCAGAGAGCCTC\|ACTGCGGCAGCTTCTCTTCTTT | +1 | HM |
| Allele 2: | TGTGATCAGAGAGCCTC\|ACTGCGGCAGCTTCTCTTCTTT | +1 |  |

**Plant SPL11-5**

Nitab_0003942g0050.1

| *gSPL3.3 seq:* | GAAATTCACAGACCTTG\|TGGCGGCGATGGTATGTC | Mut | Genotype |
| --- | --- | --- | --- |
| Allele 1: | GAAATTCACAGACCTTG\|TGGCGGCGATGGTATGTC | WT | HT |
| Allele 2: | GAAATTCACAGACCTTG\|TTGGCGGCGATGGTATGTC | +1 |  |

Nitab_0002299g0030.1

| *gSPL1.6 seq:* | ATGGACATAACAGGCGT\|CGAAGGAAACCTCAGCC | Mut | Genotype |
| --- | --- | --- | --- |
| Allele 1: | ATGGACATAACAGGCGT\|TCGAAGGAAACCTCAGCC | +1 | BA |
| Allele 2: | ATGGACATAACAGGCGT\|ACGAAGGAAACCTCAGCC | +1 |  |

Nitab_0001010g0010.1

| *gSPL1.6 seq:* | ATGGACATAACAGGCGT\|CGAAGGAAACCTCAGCC | Mut | Genotype |
| --- | --- | --- | --- |
| Allele 1: | ATGGACATAACAGGCGT\|GCGAAGGAAACCTCAGCC | +1 | HM |
| Allele 2: | ATGGACATAACAGGCGT\|GCGAAGGAAACCTCAGCC | +1 |  |

Nitab_0003900g0020.1

| *gSPL2.5 seq:* | GGAAACCATCCAGTTCA\|ACTCGGCAAGGCTTTCTTCAT | Mut | Genotype |
| --- | --- | --- | --- |
| Allele 1: | GGAAACCATCCAGTTCA\|ACTCGGCAAGGCTTTCTTCAT | WT | HT |
| Allele 2: | GGAAACCATCCAGTTCA\|AACTCGGCAAGGCTTTCTTCAT | +1 |  |

**Plant SPL11-6**

Nitab_0003942g0050.1

| *gSPL3.3 seq:* | GAAATTCACAGACCTTG\|TGGCGGCGATGGTATGTC | Mut | Genotype |
| --- | --- | --- | --- |
| Allele 1: | GAAATTCACAGACCTTG\|ATGGCGGCGATGGTATGTC | +1 | HM |
| Allele 2: | GAAATTCACAGACCTTG\|ATGGCGGCGATGGTATGTC | +1 |  |

Nitab_0004959g0040.1

| *gSPL1.3 seq:* | AAGGGTCAAGTACTTCA\|ATGAGGTGTTGCCAAGCTGA | Mut | Genotype |
| --- | --- | --- | --- |
| Allele 1: | AAGGGTCAAGTACTTCA\|ATGAGGTGTTGCCAAGCTGA | WT | HT |
| Allele 2: | AAGGGTCAAGTACTTCA\|AATGAGGTGTTGCCAAGCTGA | +1 |  |

Nitab_0002299g0030.1

| *gSPL1.6 seq:* | ATGGACATAACAGGCGT\|CGAAGGAAACCTCAGCC | Mut | Genotype |
| --- | --- | --- | --- |
| Allele 1: | ATGGACATAACAGGCGT\|TCGAAGGAAACCTCAGCC | +1 | HM |
| Allele 2: | ATGGACATAACAGGCGT\|TCGAAGGAAACCTCAGCC | +1 |  |

Nitab_0001010g0010.1

| *gSPL1.6 seq:* | ATGGACATAACAGGCGT\|CGAAGGAAACCTCAGCC | Mut | Genotype |
| --- | --- | --- | --- |
| Allele 1: | ATGGACATAACAGGCGT\|ACGAAGGAAACCTCAGCC | +1 | HM |
| Allele 2: | ATGGACATAACAGGCGT\|ACGAAGGAAACCTCAGCC | +1 |  |

Nitab_0003900g0020.1

| *gSPL2.5 seq:* | GGAAACCATCCAGTTCA\|ACTCGGCAAGGCTTTCTTCAT | Mut | Genotype |
| --- | --- | --- | --- |
| Allele 1: | GGAAACCATCCAGTTCA\|AACTCGGCAAGGCTTTCTTCAT | +1 | HM |
| Allele 2: | GGAAACCATCCAGTTCA\|AACTCGGCAAGGCTTTCTTCAT | +1 |  |

Nitab_0000861g0050.1

| *gSPL2.4 seq:* | TGTGATCAGAGAGCCTC\|CTGCGGCAGCTTCTCTTCTTT | Mut | Genotype |
| --- | --- | --- | --- |
| Allele 1: | TGTGATCAGAGAGCCTC\|ACTGCGGCAGCTTCTCTTCTTT | +1 | HM |
| Allele 2: | TGTGATCAGAGAGCCTC\|ACTGCGGCAGCTTCTCTTCTTT | +1 |  |

**Plant SPL15-1**

Nitab_0003942g0050.1

| *gSPL3.3 seq:* | GAAATTCACAGACCTTG\|TGGCGGCGATGGTATGTC | Mut | Genotype |
| --- | --- | --- | --- |
| Allele 1: | GAAATTCACAGACCT--\|TGGCGGCGATGGTATGTC | -2 | HM |
| Allele 2: | GAAATTCACAGACCT--\|TGGCGGCGATGGTATGTC | -2 |  |


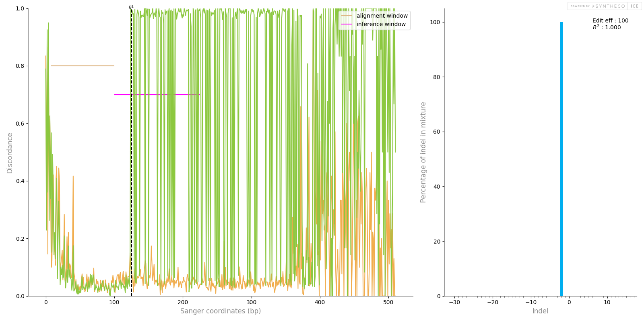

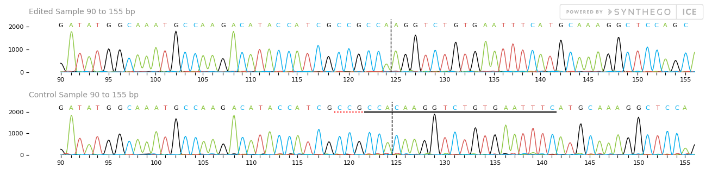


Nitab_0003348g0050.1

| *gSPL3.1 seq:* | TTAAAGGGGCATCAGTC\|CAATGGCAGAGAAATC | Mut | Genotype |
| --- | --- | --- | --- |
| Allele 1: | TTAAAGGGGCATCAGTC\|TCAATGGCAGAGAAATC | +1 | HM |
| Allele 2: | TTAAAGGGGCATCAGTC\|TCAATGGCAGAGAAATC | +1 |  |


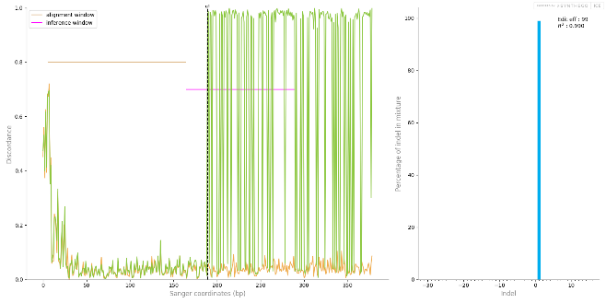

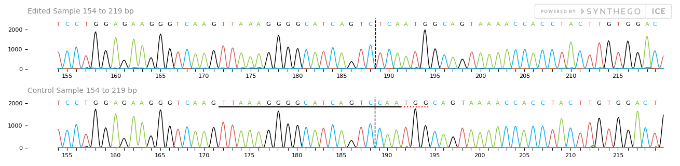


Nitab_0001538g0080.1

| *gSPL3.6 seq:* | CCTTCAACTTGACACCT\|AGGTGGCTGACCACCCT | Mut | Genotype |
| --- | --- | --- | --- |
| Allele 1: | CCTTCAACTTGACACCT\|AGGTGGCTGACCACCCT | WT | HT |
| Allele 2: | CCTTCAACTTG------\|--GTGGCTGACCACCCT | -8 |  |


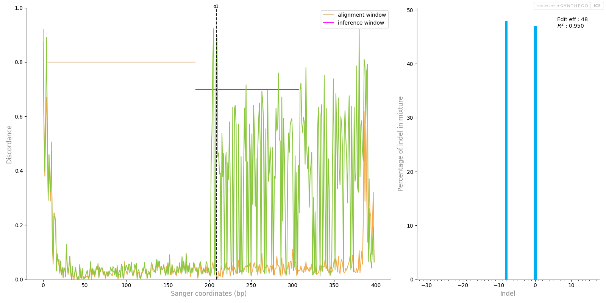

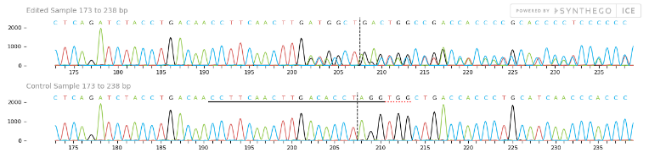


Nitab_0000991g0020.1

| *gSPL3.2 seq:* | ATCTACTTTGAAAATGT\|GGGTGGTGGGTCATCGCCGG | Mut | Genotype |
| --- | --- | --- | --- |
| Allele 1: | ATCTACTTTGAAAATGT\|GTCTCGATTGATTGAATTCTG  GTTCAAGTAAATTAGTGCTATCAGCGTACTTGGCATATG  GCTATTATGAAACTTGGGTAGCACTTTGTCCATTTGAGT  TAGTAGTTGGACTTTGGTATTGTATAATTGGGTGGTGGG  TCATCGCCGG | +128 | HM |
| Allele 2: | ATCTACTTTGAAAATGT\|GTCTCGATTGATTGAATTCTG  GTTCAAGTAAATTAGTGCTATCAGCGTACTTGGCATATG  GCTATTATGAAACTTGGGTAGCACTTTGTCCATTTGAGT  TAGTAGTTGGACTTTGGTATTGTATAATTGGGTGGTGGG  TCATCGCCGG | +128 |  |


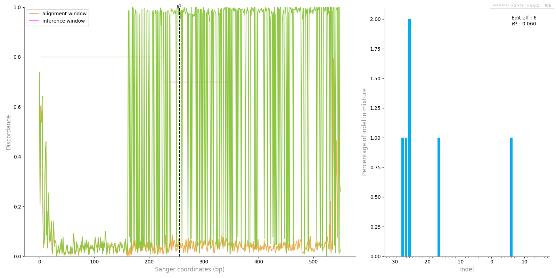

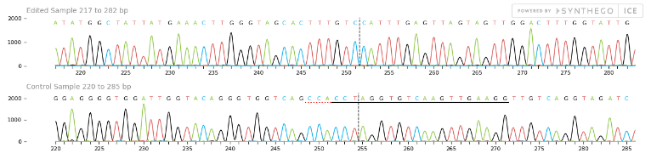


For long insertions, Synthego is not accurate predicting the type of edition. Therefore, the chromatogram was analyzed manually to determine the inserted nucleotides.


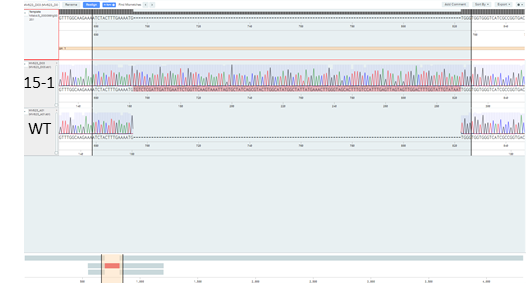


Nitab_0002299g0030.1

| *gSPL1.6 seq:* | ATGGACATAACAGGCGT\|CGAAGGAAACCTCAGCC | Mut | Genotype |
| --- | --- | --- | --- |
| Allele 1: | ATGGACATAACAGGCGT\|TCGAAGGAAACCTCAGCC | +1 | BA |
| Allele 2: | ATGGACATAACAGGCGT\|ACGAAGGAAACCTCAGCC | +1 |  |


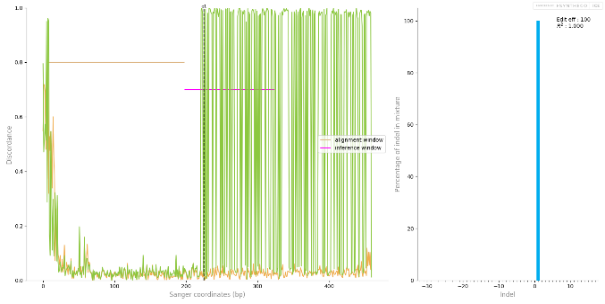

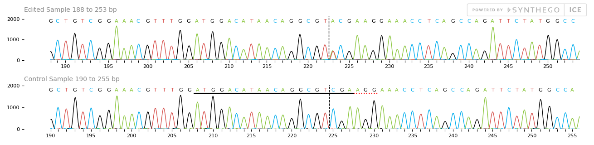


Nitab_0001010g0010.1

| *gSPL1.6 seq:* | ATGGACATAACAGGCGT\|CGAAGGAAACCTCAGCC | Mut | Genotype |
| --- | --- | --- | --- |
| Allele 1: | ATGGACATAACAGGCGT\|TCGAAGGAAACCTCAGCC | +1 | HM |
| Allele 2: | ATGGACATAACAGGCGT\|TCGAAGGAAACCTCAGCC | +1 |  |


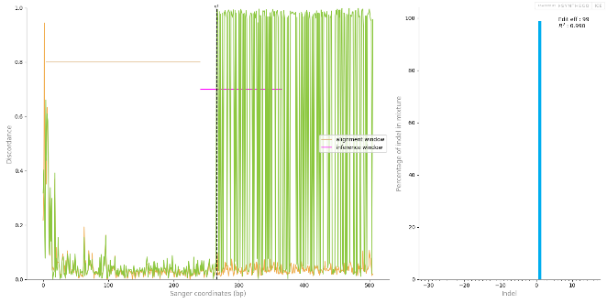

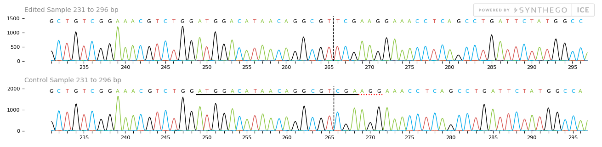


Nitab_0000067g0130.1

| *gSPL3.4 seq:* | CAGTCATTCCAAATGCC\|CAAAGGTCATCATAGGAGGTGCC | Mut | Genotype |
| --- | --- | --- | --- |
| Allele 1: | CAGTCATTCCAAAT--C\|CAAAGGTCATCATAGGAGGTGCC | -2 | HM |
| Allele 2: | CAGTCATTCCAAAT--C\|CAAAGGTCATCATAGGAGGTGCC | -2 |  |


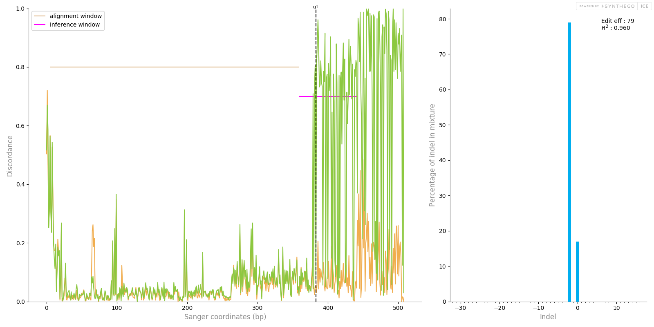

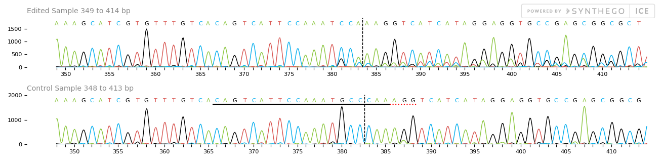


Nitab_0003900g0020.1

| *gSPL2.5 seq:* | GGAAACCATCCAGTTCA\|ACTCGGCAAGGCTTTCTTCAT | Mut | Genotype |
| --- | --- | --- | --- |
| Allele 1: | GGAAACCATCCAGTTCA\|ACTCGGCAAGGCTTTCTTCAT | WT | HT |
| Allele 2: | GGAAACCATCCAGTTCA\|AACTCGGCAAGGCTTTCTTCAT | +1 |  |


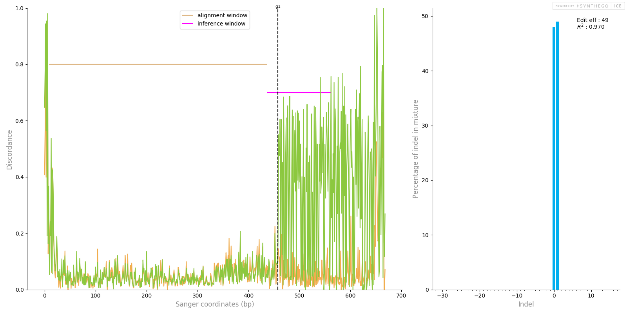

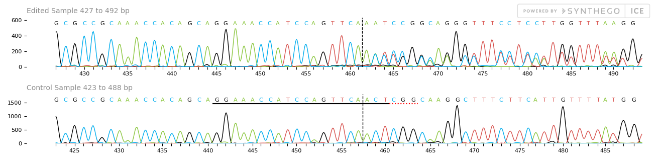


Nitab_0000861g0050.1

| *gSPL2.4 seq:* | TGTGATCAGAGAGCCTC\|CTGCGGCAGCTTCTCTTCT | Mut | Genotype |
| --- | --- | --- | --- |
| Allele 1: | TGTGATCAGAGAGCCT-\|CTGCGGCAGCTTCTCTTCT | -1 | HM |
| Allele 2: | TGTGATCAGAGAGCCT-\|CTGCGGCAGCTTCTCTTCT | -1 |  |

Due to their low quality, these chromatograms could not be analyzed using Synthego. TIDE was used for the analysis instead.


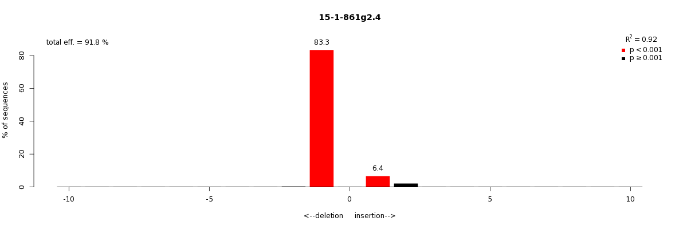

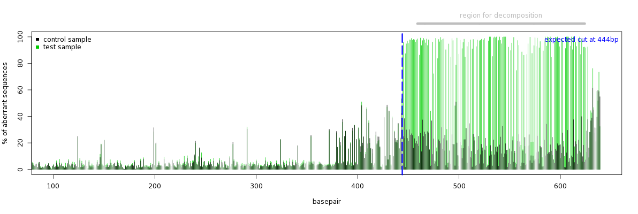


Nitab_0007217g0040.1

| *gSPL2.3 seq:* | TAGAAATTCACCCCCCA\|TGGAGGCTTTGGTAGGCT | Mut | Genotype |
| --- | --- | --- | --- |
| Allele 1: | TAGAAATTCACCCC---\|---------------GCT | -18 | HM |
| Allele 2: | TAGAAATTCACCCC---\|---------------GCT | -18 |  |


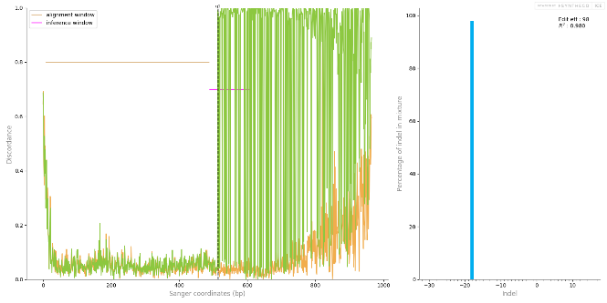

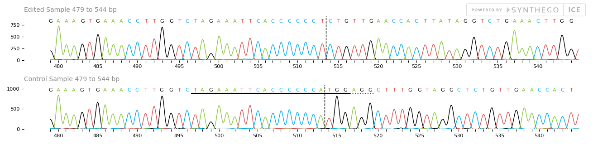


**Plant SPL22-1**

Nitab_0003942g0050.1

| *gSPL3.3 seq:* | GAAATTCACAGACCTTG\|TGGCGGCGATGGTATGTC | Mut | Genotype |
| --- | --- | --- | --- |
| Allele 1: | GAAATTCACAGACCTTG\|TTGGCGGCGATGGTATGTC | +1 | BA |
| Allele 2: | GAAATTCACAGACCTT-\|TGGCGGCGATGGTATGTC | -1 |  |
| *gSPL3.1 seq:* | TTAAAGGGGCATCAGTC\|CAATGGCAGAGAAATCAC | Mut | Genotype |
| Allele 1: | TTAAAGGGGCATCAGTC\|CAATGGCAGAGAAATCAC | WT | HT |
| Allele 2: | TTAAAGGGGCATCAGTC\|GCAATGGCAGAGAAATCAC | +1 |  |

Nitab_0003348g0050.1

| *gSPL3.1 seq:* | TTAAAGGGGCATCAGTC\|CAATGGCAGAGAAATC | Mut | Genotype |
| --- | --- | --- | --- |
| Allele 1: | TTAAAGGGGCATCAGTC\|CAATGGCAGAGAAATC | WT | HT |
| Allele 2: | TTAAAGGGGCATCAGTC\|ACAATGGCAGAGAAATC | +1 |  |

Nitab_0004959g0040.1

| *gSPL1.3 seq:* | AAGGGTCAAGTACTTCA\|ATGAGGTGTTGCCAAGCTGA | Mut | Genotype |
| --- | --- | --- | --- |
| Allele 1: | AAGGGTCAAGTACTTCA\|ATGAGGTGTTGCCAAGCTGA | WT | HT |
| Allele 2: | AAGGGTCAAGTACTTCA\|AATGAGGTGTTGCCAAGCTGA | +1 |  |
| *gSPL1.4 seq:* | CGACGGCTTGAGCCTTT\|GCATGGTATTCACAGACTTTA | Mut | Genotype |
| Allele 1: | CGACGGCTTGAGCCTTT\|GCATGGTATTCACAGACTTTA | WT | HT |
| Allele 2: | CGACGGC----------------TATTCACAGACTTTA | -16 |  |

Nitab_0001538g0080.1

| *gSPL3.6 seq:* | CCTTCAACTTGACACCT\|AGGTGGCTGACCACCCT | Mut | Genotype |
| --- | --- | --- | --- |
| Allele 1: | CCTTCAACTTGACACCT\|AGGTGGCTGACCACCCT | WT | HT |
| Allele 2: | CCTTCAACTTGACACCT\|TAGGTGGCTGACCACCCT | +1 |  |

Nitab_0002299g0030.1

| *gSPL1.6 seq:* | ATGGACATAACAGGCGT\|CGAAGGAAACCTCAGCC | Mut | Genotype |
| --- | --- | --- | --- |
| Allele 1: | ATGGACATAACAGGCGT\|ACGAAGGAAACCTCAGCC | +1 | BA |
| Allele 2: | ATGGACATAACAGGC-T\|CGAAGGAAACCTCAGCC | -1 |  |

Nitab_0001010g0010.1

| *gSPL1.6 seq:* | ATGGACATAACAGGCGT\|CGAAGGAAACCTCAGCC | Mut | Genotype |
| --- | --- | --- | --- |
| Allele 1: | ATGGACATAACAGGCGT\|TCGAAGGAAACCTCAGCC | +1 | BA |
| Allele 2: | ATGGACATAACAGGCGT\|ACGAAGGAAACCTCAGCC | +1 |  |

Nitab_0003900g0020.1

| *gSPL3.4 seq:* | CAGTCATTCCAAATGCC\|CAAAGGTCATTATAGCAGG | Mut | Genotype |
| --- | --- | --- | --- |
| Allele 1: | CAGTCATTCCAAATGCC\|CAAAGGTCATTATAGCAGG | WT | HT |
| Allele 2: | CAGTCATTCCAAATGC-\|CAAAGGTCATTATAGCAGG | -1 |  |
| *gSPL2.5 seq:* | GGAAACCATCCAGTTCA\|ACTCGGCAAGGCTTTCTTCAT | Mut | Genotype |
| Allele 1: | GGAAACCATCCAGTTCA\|ACTCGGCAAGGCTTTCTTCAT | WT | HT |
| Allele 2: | GGAAACCATCCAGTTCA\|AACTCGGCAAGGCTTTCTTCAT | +1 |  |
| *gSPL3.5 seq:* | AGATGGAGTGGAATGCA\|AAGTGGGACTGGGGAAACCTGG | Mut | Genotype |
| Allele 1: | AGATGGAGTGGAATGCA\|AAGTGGGACTGGGGAAACCTGG | WT | HT |
| Allele 2: | AGATGGAGTGGAAT---\|AAGTGGGACTGGGGAAACCTGG | -3 |  |

Nitab_0000861g0050.1

| *gSPL2.4 seq:* | TGTGATCAGAGAGCCTC\|CTGCGGCAGCTTCTCTTCTTT | Mut | Genotype |
| --- | --- | --- | --- |
| Allele 1: | TGTGATCAGAGAGCCT-\|CTGCGGCAGCTTCTCTTCTTT | -1 | HM |
| Allele 2: | TGTGATCAGAGAGCCT-\|CTGCGGCAGCTTCTCTTCTTT | -1 |  |
| *gSPL2.3 seq:* | TAGAAATTCACCCCCCA\|TGGAGGCTCCAGTAGGCTCTGT | Mut | Genotype |
| Allele 1: | TAGAAATTCACCCCCCA\|TGGAGGCTCCAGTAGGCTCTGT | WT | HT |
| Allele 2: | TAGAAATTCACCCCCCA\|ATGGAGGCTCCAGTAGGCTCTGT | +1 |  |

**Plant SPL22-2**

Nitab_0003942g0050.1

| *gSPL3.3 seq:* | GAAATTCACAGACCTTG\|TGGCGGCGATGGTATGTC | Mut | Genotype |
| --- | --- | --- | --- |
| Allele 1: | GAAATTCACAGACCTTG\|TTGGCGGCGATGGTATGTC | +1 | BA |
| Allele 2: | GAAATTCACAGACCTT-\|TGGCGGCGATGGTATGTC | -1 |  |
| *gSPL3.1 seq:* | TTAAAGGGGCATCAGTC\|CAATGGCAGAGAAATCAC | Mut | Genotype |
| Allele 1: | TTAAAGGGGCATCAGTC\|CAATGGCAGAGAAATCAC | WT | HT |
| Allele 2: | TTAAAGGGGCATCAGTC\|GCAATGGCAGAGAAATCAC | +1 |  |

Nitab_0003348g0050.1

| *gSPL3.1 seq:* | TTAAAGGGGCATCAGTC\|CAATGGCAGAGAAATC | Mut | Genotype |
| --- | --- | --- | --- |
| Allele 1: | TTAAAGGGGCATCAGTC\|CAATGGCAGAGAAATC | WT | HT |
| Allele 2: | TTAAAGGGGCATCAGTC\|ACAATGGCAGAGAAATC | +1 |  |

Nitab_0004959g0040.1

| *gSPL1.3 seq:* | AAGGGTCAAGTACTTCA\|ATGAGGTGTTGCCAAGCTGA | Mut | Genotype |
| --- | --- | --- | --- |
| Allele 1: | AAGGGTCAAGTACTTCA\|AATGAGGTGTTGCCAAGCTGA | +1 | HM |
| Allele 2: | AAGGGTCAAGTACTTCA\|AATGAGGTGTTGCCAAGCTGA | +1 |  |
| *gSPL1.4 seq:* | CGACGGCTTGAGCCTTT\|GCATGGTATTCACAGACTTTA | Mut | Genotype |
| Allele 1: | CGACGGC----------------TATTCACAGACTTTA | -16 | HM |
| Allele 2: | CGACGGC----------------TATTCACAGACTTTA | -16 |  |

Nitab_0001538g0080.1

| *gSPL3.6 seq:* | CCTTCAACTTGACACCT\|AGGTGGCTGACCACCCT | Mut | Genotype |
| --- | --- | --- | --- |
| Allele 1: | CCTTCAACTTGACACCT\|AGGTGGCTGACCACCCT | WT | HT |
| Allele 2: | CCTTCAACTTGACACCT\|TAGGTGGCTGACCACCCT | +1 |  |

Nitab_0002299g0030.1

| *gSPL1.6 seq:* | ATGGACATAACAGGCGT\|CGAAGGAAACCTCAGCC | Mut | Genotype |
| --- | --- | --- | --- |
| Allele 1: | ATGGACATAACAGGCGT\|ACGAAGGAAACCTCAGCC | +1 | BA |
| Allele 2: | ATGGACATAACAGGC-T\|CGAAGGAAACCTCAGCC | -1 |  |

Nitab_0001010g0010.1

| *gSPL1.6 seq:* | ATGGACATAACAGGCGT\|CGAAGGAAACCTCAGCC | Mut | Genotype |
| --- | --- | --- | --- |
| Allele 1: | ATGGACATAACAGGCGT\|TCGAAGGAAACCTCAGCC | +1 | HM |
| Allele 2: | ATGGACATAACAGGCGT\|TCGAAGGAAACCTCAGCC | +1 |  |

Nitab_0003900g0020.1

| *gSPL3.4 seq:* | CAGTCATTCCAAATGCC\|CAAAGGTCATTATAGCAGG | Mut | Genotype |
| --- | --- | --- | --- |
| Allele 1: | CAGTCATTCCAAATGCC\|CAAAGGTCATTATAGCAGG | WT | HT |
| Allele 2: | CAGTCATTCCAAATGC-\|CAAAGGTCATTATAGCAGG | -1 |  |
| *gSPL2.5 seq:* | GGAAACCATCCAGTTCA\|ACTCGGCAAGGCTTTCTTCAT | Mut | Genotype |
| Allele 1: | GGAAACCATCCAGTTCA\|ACTCGGCAAGGCTTTCTTCAT | WT | HT |
| Allele 2: | GGAAACCATCCAGTTCA\|AACTCGGCAAGGCTTTCTTCAT | +1 |  |
| *gSPL3.5 seq:* | AGATGGAGTGGAATGCA\|AAGTGGGACTGGGGAAACCTGG | Mut | Genotype |
| Allele 1: | AGATGGAGTGGAATGCA\|AAGTGGGACTGGGGAAACCTGG | WT | HT |
| Allele 2: | AGATGGAGTGGAAT---\|AAGTGGGACTGGGGAAACCTGG | -3 |  |

Nitab_0000861g0050.1

| *gSPL2.4 seq:* | TGTGATCAGAGAGCCTC\|CTGCGGCAGCTTCTCTTCTTTT | Mut | Genotype |
| --- | --- | --- | --- |
| Allele 1: | TGTGATCAGAGAGCCT-\|CTGCGGCAGCTTCTCTTCTTTT | -1 | HM |
| Allele 2: | TGTGATCAGAGAGCCT-\|CTGCGGCAGCTTCTCTTCTTTT | -1 |  |
| *gSPL2.3 seq:* | TAGAAATTCACCCCCCA\|TGGAGGCTCCAGTAGGCTCTGTT | Mut | Genotype |
| Allele 1: | TAGAAATTCACCCCCCA\|ATGGAGGCTCCAGTAGGCTCTGTT | +1 | HM |
| Allele 2: | TAGAAATTCACCCCCCA\|ATGGAGGCTCCAGTAGGCTCTGTT | +1 |  |

**Plant SPL22-6**

Nitab_0003942g0050.1

| *gSPL3.3 seq:* | GAAATTCACAGACCTTG\|TGGCGGCGATGGTATGTC | Mut | Genotype |
| --- | --- | --- | --- |
| Allele 1: | GAAATTCACAGACCTT-\|TGGCGGCGATGGTATGTC | -1 | HM |
| Allele 2: | GAAATTCACAGACCTT-\|TGGCGGCGATGGTATGTC | -1 |  |

Nitab_0003348g0050.1

| *gSPL3.1 seq:* | TTAAAGGGGCATCAGTC\|CAATGGCAGAGAAATC | Mut | Genotype |
| --- | --- | --- | --- |
| Allele 1: | TTAAAGGGGCATCAGTC\|ACAATGGCAGAGAAATC | +1 | HM |
| Allele 2: | TTAAAGGGGCATCAGTC\|ACAATGGCAGAGAAATC | +1 |  |

Nitab_0004959g0040.1

| *gSPL1.3 seq:* | AAGGGTCAAGTACTTCA\|ATGAGGTGTTGCCAAGCTGA | Mut | Genotype |
| --- | --- | --- | --- |
| Allele 1: | AAGGGTCAAGTACTTCA\|AATGAGGTGTTGCCAAGCTGA | +1 | HM |
| Allele 2: | AAGGGTCAAGTACTTCA\|AATGAGGTGTTGCCAAGCTGA | +1 |  |
| *gSPL1.4 seq:* | CGACGGCTTGAGCCTTT\|GCATGGTATTCACAGACTTTA | Mut | Genotype |
| Allele 1: | CGACGGC----------------TATTCACAGACTTTA | -16 | HM |
| Allele 2: | CGACGGC----------------TATTCACAGACTTTA | -16 |  |

Nitab_0001538g0080.1

| *gSPL3.6 seq:* | CCTTCAACTTGACACCT\|AGGTGGCTGACCACCCT | Mut | Genotype |
| --- | --- | --- | --- |
| Allele 1: | CCTTCAACTTGACACCT\|AGGTGGCTGACCACCCT | WT | HT |
| Allele 2: | CCTTCAACTTGACACCT\|TAGGTGGCTGACCACCCT | +1 |  |

Nitab_0002299g0030.1

| *gSPL1.6 seq:* | ATGGACATAACAGGCGT\|CGAAGGAAACCTCAGCC | Mut | Genotype |
| --- | --- | --- | --- |
| Allele 1: | ATGGACATAACAGGCGT\|ACGAAGGAAACCTCAGCC | +1 | BA |
| Allele 2: | ATGGACATAACAGGC-T\|CGAAGGAAACCTCAGCC | -1 |  |

Nitab_0001010g0010.1

| *gSPL1.6 seq:* | ATGGACATAACAGGCGT\|CGAAGGAAACCTCAGCC | Mut | Genotype |
| --- | --- | --- | --- |
| Allele 1: | ATGGACATAACAGGCGT\|ACGAAGGAAACCTCAGCC | +1 | HM |
| Allele 2: | ATGGACATAACAGGCGT\|ACGAAGGAAACCTCAGCC | +1 |  |

Nitab_0003900g0020.1

| *gSPL3.4 seq:* | CAGTCATTCCAAATGCC\|CAAAGGTCATTATAGCAGG | Mut | Genotype |
| --- | --- | --- | --- |
| Allele 1: | CAGTCATTCCAAATGCC\|CAAAGGTCATTATAGCAGG | WT | HT |
| Allele 2: | CAGTCATTCCAAATGC-\|CAAAGGTCATTATAGCAGG | -1 |  |
| *gSPL2.5 seq:* | GGAAACCATCCAGTTCA\|ACTCGGCAAGGCTTTCTTC | Mut | Genotype |
| Allele 1: | GGAAACCATCCAGTTCA\|ACTCGGCAAGGCTTTCTTC | WT | HT |
| Allele 2: | GGAAACCATCCAGTTCA\|AACTCGGCAAGGCTTTCTTC | +1 |  |
| *gSPL3.5 seq:* | AGATGGAGTGGAATGCA\|AAGTGGGACTGGGGAAACC | Mut | Genotype |
| Allele 1: | AGATGGAGTGGAATGCA\|AAGTGGGACTGGGGAAACC | WT | HT |
| Allele 2: | AGATGGAGTGGAAT---\|AAGTGGGACTGGGGAAACC | -3 |  |

Nitab_0000861g0050.1

| *gSPL2.4 seq:* | TGTGATCAGAGAGCCTC\|CTGCGGCAGCTTCTCTTCT | Mut | Genotype |
| --- | --- | --- | --- |
| Allele 1: | TGTGATCAGAGAGCCT-\|CTGCGGCAGCTTCTCTTCT | -1 | HM |
| Allele 2: | TGTGATCAGAGAGCCT-\|CTGCGGCAGCTTCTCTTCT | -1 |  |
